# Is Therapeutic Drug Monitoring Relevant for Antidepressant Drug Therapy? Implications From a Systematic Review and Meta-Analysis With Focus on Moderating Factors

**DOI:** 10.3389/fpsyt.2022.826138

**Published:** 2022-02-21

**Authors:** Cleo S. M. Funk, Xenia M. Hart, Gerhard Gründer, Christoph Hiemke, Björn Elsner, Reinhold Kreutz, Thomas G. Riemer

**Affiliations:** ^1^Department of Psychology, Humboldt-Universität zu Berlin, Berlin, Germany; ^2^Department of Molecular Neuroimaging, Central Institute of Mental Health, Medical Faculty Mannheim, University of Heidelberg, Mannheim, Germany; ^3^Department of Psychiatry and Psychotherapy, University Medical Center, Mainz, Germany; ^4^Charité – Universitätsmedizin Berlin, Institute of Clinical Pharmacology and Toxicology, Berlin, Germany

**Keywords:** therapeutic drug monitoring (TDM), depression, concentration, meta-regression, efficacy, moderators

## Abstract

Inter-individual differences in antidepressant drug concentrations attained in blood may limit the efficacy of pharmacological treatment of depressive disorders. Therapeutic drug monitoring (TDM) enables to determine drug concentrations in blood and adjust antidepressant dosage accordingly. However, research on the underlying assumption of TDM, association between concentration and clinical effect, has yielded ambiguous results for antidepressants. It has been proposed that this ambiguity may be caused by methodological shortcomings in studies investigating the concentration-effect relationship. Guidelines recommend the use of TDM in antidepressant treatment as expert opinion. This reflects the lack of research, particularly systematic reviews and meta-analyses of randomized controlled trials, on the relationship between concentration and effect as well as on the benefits of the use of TDM in clinical practice. In this study, a systematic review and meta-analysis of randomized controlled trials has been performed to investigate the relationship between antidepressant concentration, efficacy, and side effects. It is the first meta-analytical approach to this subject and additionally considers methodological properties of primary studies as moderators of effect in quantitative analysis. Our results identified methodological shortcomings, namely the use of a flexible dose design and the exclusion of concentrations in lower- or subtherapeutic ranges, which significantly moderate the relationship between antidepressant concentration and efficacy. Such shortcomings obscure the evidence base of using TDM in clinical practice to guide antidepressant drug therapy. Further research should consider these findings to determine the relationship between concentration and efficacy and safety of antidepressant treatments, especially for newer antidepressants.

**Systematic Review Registration:**

https://www.crd.york.ac.uk/prospero/display_record.php?RecordID=246149, identifier: CRD42021246149.

## Introduction

Depressive disorders are among the most frequent and disabling diseases worldwide. Estimates on the 12-month prevalence indicate a global percentage of 3.76% across all age groups and sexes (1). Among psychiatric illnesses, depressive disorders account for the highest number of disability-adjusted life years (DALYs) (1). Treatment recommendations for depressive disorders include psychotherapy and psychopharmacotherapy. There is substantial evidence for the efficacy of antidepressant drug treatment (2), which has been confirmed in a recent extensive meta-analysis (3). However, numbers on the response rates in trials have been estimated at only 50–60% (4). Furthermore, estimates on treatment adherence vary greatly between 40 and 90% (5). To enhance treatment efficacy, methods have been developed to consider the high degree of individuality in antidepressant treatment, focusing on the individual situation of patients. These methods are referred to collectively as “precision medicine” or “personalized medicine” (6). One aspect that has been highlighted in antidepressant research is a substantial pharmacokinetic variability of antidepressant medications. For several substances, concentrations in blood have been shown to vary more than 20-fold between individuals (7). Therapeutic drug monitoring (TDM), as a tool of precision medicine, considers this variability by measuring drug concentrations in blood (serum or plasma) and thus provides guidance for individualized dosing strategies. TDM is applied in routine care in the treatment of multiple somatic diseases, including epilepsy, infectious diseases, and cardiovascular diseases, as well as following organ transplantation (8). In psychiatric care, TDM is compulsory for lithium treatment, due to the narrow therapeutic range and the risk for severe adverse effects (9). Regarding antidepressant agents, recommendations for TDM have been stated for tricyclic antidepressants (TCAs) due to their cardiotoxic potential (9). Notably, this recommendation rather aims at improving treatment safety than efficacy.

However, expert based guidelines for TDM use in antidepressant treatment do not recommend TDM as standard of care (2). The reason for this is limited evidence on the basic assumption of TDM, namely an association between concentration and clinical effects for both, treatment efficacy and safety (10). For TCAs, systematic reviews were able to demonstrate a significant relationship between concentration and clinical improvement (11, 12). The situation differs profoundly for selective serotonin reuptake-inhibitors (SSRIs) and selective serotonin-noradrenaline reuptake-inhibitors (SSNRIs), the most frequently prescribed drugs in the treatment of depression today (13). Mitchell (14) reviewed research on a potential pharmacokinetic/pharmacodynamic (PK-PD) relationship in non-tricyclic antidepressants, summarizing that it was not possible to obtain reliable evidence for a significant relationship between concentration and clinical effects. Other reviews investigating a potential association between antidepressant concentration and clinical effects have reported similar conclusions, with affirmations for an association between clinical outcomes with TCAs and heterogeneous findings in the investigation of SSRIs/SSNRIs and other non-tricyclic antidepressants (15–21). An overview of reviews on the subject from 1977 to today is given in Supplementary Table 1.

Notably, conclusions which can be drawn from reviews and meta-analyses strongly depend on the quality of primary studies. It has been argued that methodological shortcomings in primary studies may account for the heterogeneity of evidence on the PK-PD relationship of antidepressant drugs (22). Systematic approaches to identify potentially relevant methodological shortcomings have been proposed by several authors (22–24). Suggested areas of scrutiny were assessment of clinical outcomes, patient characteristics, study design, concentration design, and dose design. The present study systematically reviewed and analyzed randomized controlled trials (RCTs) which provided information on the concentration-effect relationship (efficacy or tolerability) in antidepressant treatment. We aimed at addressing the question whether there is an association between antidepressant drug concentration and clinical effects (efficacy and side effects) and identified methodological shortcomings in primary studies that systematically influence the relationship between the variables. These questions will be approached both qualitatively and quantitatively. The present study is, to our knowledge, the first meta-analysis investigating the relationship between antidepressant concentration and efficacy.

## Methods

### Study Selection Process

The updated PRISMA criteria (25) were followed for the present study. In a first step, the PubMed and Web of Science databases were searched for RCTs as well as reviews and meta-analyses (search last updated November 5, 2021). Search algorithms included keywords for antidepressant drugs, therapeutic drug monitoring, and concentration as well as indicators for randomized trials or reviews/meta-analyses (Supplementary Table 2). Records were screened by two independent raters (TGR and CSMF). References from eligible RCTs and reviews/meta-analyses were screened for further suitable records by the same raters. The study was registered with PROSPERO (ID: CRD42021246149). Whenever studies were eligible but data on concentration values was not available, data was requested from the authors. Criteria for inclusion and exclusion according to PICOS (26) are listed in Table 1.

**Table 1:** Study selection process: inclusion and exclusion criteria (PICOS).

**Domain**	**Criteria**	
	**Inclusion**	**Exclusion**
Population	Patients with depressive disorders (Major depression or dysthymia), studies including small numbers of bipolar patients currently being treated for depressive symptoms were also eligible	Patients fulfilling criteria for psychotic features
Intervention	Patients treated with antidepressant medication as specified in the search algorithm	Treatment with currently not recommended antidepressant medication (9)
Comparison/ Control group	Active, placebo, dose groups, concentration range groups	Augmentation studies without study arm without augmentation
Outcomes	Relationship between antidepressant concentration and clinical effect, either efficacy, side effects, or both; qualitative and quantitative report were acceptable	Graphic display of concentration-effect relationships without qualitative or quantitative explanation in study
Study Design	Randomized controlled trials	Non-randomized studies, randomized cross-over studies, long-term follow-up studies, short-term intravenous treatment arms

### Risk of Bias Rating and Reporting Bias Assessment

All eligible studies were assessed with the Cochrane Risk of Bias tool 2.0 (27) by two independent raters (XMH and CSMF). Disagreement was resolved through discussion. Results were visualized using robvis (28). Reporting bias was assessed by screening ClinicalTrials.gov for potentially unpublished records with the search term “depression” in conjunction with either “therapeutic drug monitoring”, “plasma”, “serum”, or “blood” (search last updated November 9, 2021). To account for possible publication bias, records included in the meta-analysis were inspected visually *via* funnel plot and evaluated statistically by linear regression test of funnel plot asymmetry (29) performed in R version 4.1.1 (30) using the metabias function from the meta package (31).

### Data Extraction

#### Outcome Definition

Outcomes of interest were reports of an association between antidepressant concentration and clinical effect, either efficacy or side effects. Reports could be qualitative or quantitative. Continuous as well as categorical associations were eligible; graphical presentation without in-text explanation or statistical presentation were not eligible.

#### Qualitative Synthesis

Outcome criteria for qualitative synthesis were extracted from eligible studies, separately for each substance included in studies if available.

#### Quantitative Synthesis

For quantitative synthesis, means and standard deviations of antidepressant concentrations in responders and non-responders were assessed. Data on antidepressant concentration was either reported in studies or calculated manually in cases where numbers for concentrations and clinical response assessments for the whole sample were given. Two a priori decisions were made in the process of quantitative data extraction: Firstly, criteria for response were taken from the primary study if given or set to a response criterion suitable for the instrument used for the determination of depression severity. This criterion was used for all studies employing the same instrument. Secondly, if studies reported concentrations from multiple assessments over the course of the study, it was decided to differentiate between fixed dose and flexible dose studies. In fixed dose studies, concentration values after 2 weeks of consecutive treatment were used for the analysis. In flexible dose studies, the last report of concentration values in the study was taken. Concentration values for drugs were calculated as sum of all active compounds, as defined by Hiemke et al. (9).

#### Concentration-Effect Specific Quality Assessment

To investigate the impact of potential moderators in antidepressant concentration-effect research, definitions of moderators and their operationalization had to be made. To validate previous research on the influence of methodological shortcomings of primary studies as moderators of the antidepressant concentration-effect relationship, proposed criteria from three studies were included (22–24) and adapted to the specific purpose of this study. The inclusion of these criteria has also been recommended in a recent protocol guideline for systematic reviews for the development of therapeutic reference ranges in psychotropic drugs (32). In total, 14 criteria were formulated to assess the quality of primary studies, shown in detail in Table 2. These criteria pertain to the assessment of clinical outcomes, patient level characteristics, study design, concentration design, and dose design. Outcome criteria extraction in qualitative and quantitative synthesis as well as rating of 14 quality criteria were performed for treatment arms rather than studies, since treatment arms could differ in report of outcome criteria and quality assessment. The quality assessment was performed by two independent raters (CSMF and TGR). Due to the broad range of publication dates of studies included in this review and the potential confounding of quality assessment results and age of publication, a table of quality assessment results by decade of publication was produced.

**Table 2:** Quality assessment criteria.

**Criteria numbers and names**		**Definition/Operationalization**
1.	Validated analytical method for the determination of concentration in serum or plasma	Chromatography (all substances) or immunoassay (TCAs only) (4, 23).
2.	Steady-state	Rated sufficient, if reported by authors.
3.	Blood sampling and drug intake described	Rated sufficient, if reported by authors and sampling time of concentrations was given.
4.	Patient selection	Classification system and diagnosis provided.
5.	Measurement of illness severity and registration of therapeutic improvement or worsening: structured scale	Rated sufficient if severity assessments were performed using structured scales.
6.	Baseline assessment of depression severity	Only if numerically reported, graphic display was not considered sufficient.
7.	Adequate calculation of change	Percent improvement, predefined response-criterion, baseline-final score, Δ baseline-final score.
8.	Sufficient time to rate effect	Minimum treatment duration: 2 weeks.
9.	Comedication	Rated sufficient, if no comedication, which could influence pharmacokinetic or pharmacodynamic properties, is given, or if a sub-analysis is provided. Exception was constant somatic pre-treatment medication.
10.	Placebo run-in	Placebo wash-out or run-in phase at the beginning of treatment.
11.	Elimination of placebo responders	Elimination of placebo responders after predefined response criterion.
12.	Dose design: fixed vs. flexible dose	Fixed dose design required for at least 2 weeks, exception: Titration in first 2 weeks of treatment. Otherwise rated insufficient.
13.	Lower concentrations included to avoid ceiling effects (**±**10%)	Reported concentration means **±** SD or range include lower part of therapeutic reference ranges (**±**10% of minimum concentration), graphic display not sufficient.
14.	Adequate quantification of Side effects: structured scale or objective measurement	Rated sufficient, if a structured scale or objective measurement (e.g., Electrocardiogram, blood pressure, or pulse measurement) was used for assessment.

*Item 13 therapeutic reference ranges see Hiemke et al. (9)*.

### Statistical Analysis

#### Overall Effect

To quantitatively investigate the relationship between antidepressant concentration and efficacy, an overall meta-analysis of differences in the antidepressant concentration between responders and non-responders was conducted *via* RevMan (Version 5.4.1) (33) using standardized mean differences and Hedges' g as effect estimate in a random effects model to account for assumed between-study heterogeneity. I^2^ statistic was used for the assessment of heterogeneity in effect sizes. Unfortunately, the relationship between antidepressant concentration and side effects could not be investigated quantitatively, since the methods of side effects assessment and reporting in primary studies were highly heterogeneous.

### Sensitivity Analyses

Sensitivity analyses were performed according to the following a priori defined criteria: Exclusion of the study with the highest weight, exclusion of studies including bipolar patients, exclusion of studies not adopting a 50% response criterion, exclusion of studies not using the Hamilton Depression Rating Scale (HAMD) (34) or the Montgomery-Asberg Depression Rating Scale (MADRS) (35) as assessment of depression severity, exclusion of studies exhibiting a high risk of bias, exclusion of studies not investigating a concentration-efficacy relationship as primary endpoint, and exclusion of studies with mean concentrations outside substance-specific therapeutic reference ranges as defined by current guidelines (9).

### Analysis of Quality Assessment Criteria as Moderators

Quantitative analyses were performed in a stepwise exploratory procedure. To quantify the impact of quality assessment criteria as moderators of a concentration-efficacy relationship, a three-step procedure was applied: The first two steps have been described recently by Harrer et al. (36) for the execution of meta-analyses in R.

I. First, a forced-entry meta-regression was performed using a mixed effects model and maximum likelihood effect estimator. The maximum likelihood effect estimator was chosen over a restricted maximum likelihood effect estimator to allow calculation of ANOVAs at a later point in the analysis. The effect sizes included in the meta-regression were also calculated in R and were compared to those computed *via* RevMan to ensure equivalency. Predictors included in the meta-regression were dichotomous ratings of twelve quality assessment criteria in each treatment arm as either “sufficient” (1) or “insufficient” (0). Criteria 5 (*Structured scale*) and 14 (*Adequate quantification of side effects*) were not included due to redundancy or inapplicability to the specific investigation of concentration-efficacy relationships.
II. Afterwards, to test the specific contribution of single quality assessment criteria as predictors, an iterative approach was applied, calculating ANOVAs sequentially including predictors in the model. Model fit was evaluated *via* likelihood ratio test. Predictors reaching at least trend-level (*p* ≤ 0.1) were included in further analyses. Both the meta-regression and the ANOVAs were calculated in R (30) using meta and metafor packages (31, 37).
III. To investigate whether the quality assessment criteria identified as relevant by this procedure would account for differences in meta-analytical effect estimates, subgroup analyses were performed *via* RevMan comparing studies rated sufficient or insufficient on each of those criteria. Subgroup comparisons were only conducted if a minimum of three records per subgroup were available and the predictor reached at least trend-level (*p* ≤ 0.1) in the preceding analyses.

Finally, forest plots of subgroup differences identified as significant (*p* ≤ 0.05) were retrieved for visualization of subgroup differences.

The influence of subgroup variables identified as significant in the total sample was re-evaluated in the smaller subsamples of TCAs and SSRIs.

### Further Subgroup Analyses

Additional subgroup analyses were conducted with RevMan to investigate the influence of participant age (38, 39), antidepressant classes, cumulative study quality, and publication date (before 1990 and after 1990). To investigate cumulative study quality, studies were grouped according to median split quality assessment (23, 24). The cut-off for publication date before and after 1990 was considered as subgroup variable, because it has been argued that placebo rates in antidepressant treatment studies have been stable since the 1990s (40). In addition, a subgroup analysis for placebo- vs. active-controlled trials was considered but not possible, as only two placebo-controlled studies were eligible for meta-analysis.

## Results

### Study Selection Process

The study selection process is presented in detail in Supplementary Figure 1. Out of 4,934 records, 170 were suitable for full-text analysis. A total of 65 studies encompassing 3,782 participants was found eligible for inclusion in qualitative synthesis. Of these, 19 studies, providing data on 764 participants, were included in the meta-analysis. The majority of studies had an active control-group design (*N* = 40), followed by dose control-group design (*N* = 9) and placebo control-group design (*N* = 8). Three studies included an augmentation control-group design, and two compared concentration groups. One study each had an active plus dose control-group design, TDM vs. no TDM, and traditional vs. intravenous dosing design, respectively. Substance classes included in this review were TCAs (47 studies, 57 treatment arms), SSRIs (21 studies, 27 treatment arms), tetracyclic antidepressants (9 studies, 9 treatment arms), SSNRIs (6 studies, 6 treatment arms), one MAO inhibitor (moclobemide; one study, one treatment arm), and one selective noradrenaline-dopamine reuptake inhibitor (bupropion; one study, one treatment arm). Key information for included substances is summarized in Supplementary Table 3, including relevant enzymes and efflux transporters involved in substance metabolization, adverse effects associated with the substance classes, and usual daily dose. In addition, information relevant for TDM is given, including elimination half-life, therapeutic reference ranges, and levels of recommendation for TDM as reported by Hiemke et al. (9).

The most common reasons for exclusion were the absence of a report or insufficient information on antidepressant concentration-effect relationships and unsuitable study design or sample characteristics. As studies often included multiple active comparators, the total number of treatment arms was 101 in the qualitative synthesis and 27 in the quantitative synthesis. Detailed information on all included trials with antidepressant drug, number of subjects with concentration data, percentage of female participants, mean age, diagnosis and classification system, dose of antidepressant drug, control group, outcomes (efficacy and side effects), and results from quality assessment are shown in Supplementary Table 4. This study does not include unpublished data.

### Risk of Bias Rating and Reporting Bias Assessment

Fifty studies exhibited a high risk of bias in at least one of the domains (Supplementary Figure 2). Only one study was rated with a low risk of bias. Linear regression test of funnel plot asymmetry as well as visual inspection did not exhibit significant results (*t* = 0.81, *p* = 0.42) (Supplementary Figure 3). Search for unpublished records on ClinicalTrials.gov yielded one potentially eligible study (identifier NCT00812812). The record was not published; results were reported but did not fulfill inclusion criteria for this review.

### Qualitative Synthesis

A summary of the results is provided in Table 3. In general, the reports of studies on concentration-efficacy relationships are highly heterogeneous, with a majority of studies reporting no relationship between the variables. However, in the subsamples of studies investigating TCAs and SSRIs, more studies report findings of a positive association between concentration and efficacy (higher concentrations associated with greater response) than of a negative association (higher concentrations associated with poorer response). The majority of studies reporting information on concentration-side effects relationships likewise reported no association between concentration and side effects. Nevertheless, studies which did find an association between concentration and side effects unanimously report positive relationships, indicating more frequent or more severe side effects with higher concentrations.

**Table 3:** Summarized results of the qualitative synthesis of 101 treatment arms in 65 studies.

	**Concentration-efficacy**			**Concentration-side effects**		
**Substance class (*N* studies, *N* treatment arms)**	**Positive C-E relationship**	**Negative C-E relationship**	**No C-E relationship**	**Positive C-SE relationship**	**Negative C-SE relationship**	**No C-SE relationship**
TCA (47, 57)	19	5	33	9	–	14
SSRI (21, 27)	5	2	20	5	–	9
Tetra-CA (9, 9)	1	1	7	1	–	4
SSNRI (6, 6)	1	–	5	2	–	–
MAO-Inhibitors (1, 1)	–	–	1	–	–	–
SNDRI (1, 1)	–	–	1	–	–	–

*C-E, Concentration-efficacy; C-SE, Concentration-side effects; SSNRI, Venlafaxine; MAO-Inhibitors, Moclobemide; SNDRI, Bupropion*.

### Quantitative Synthesis

**Figure 1:**
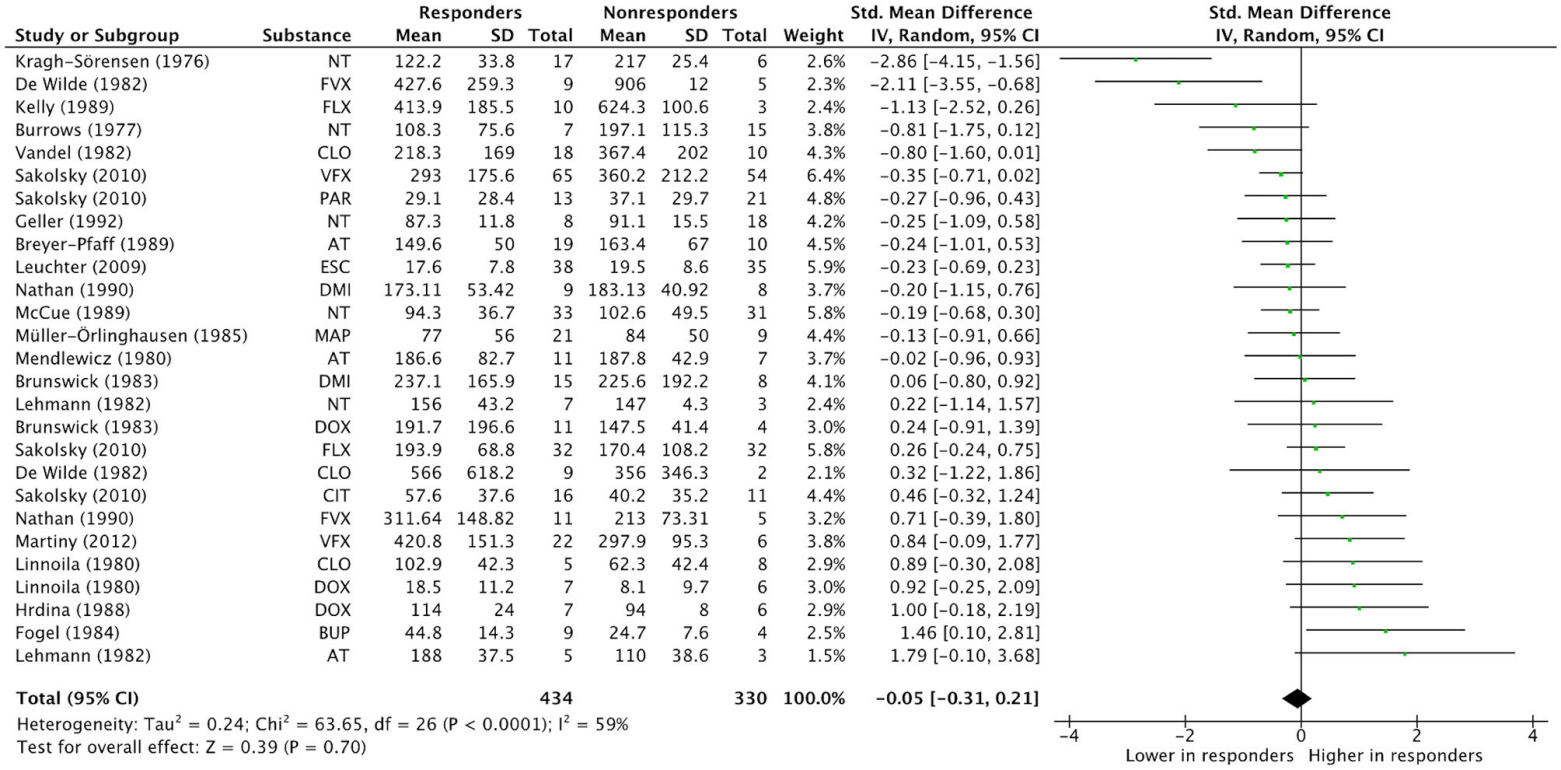
Overall effect estimate. Overall effect estimates across N treatment arms = 27, in N = 19 studies. AT, Amitriptyline; BUP, Bupropion; CIT, Citalopram; CLO, Clomipramine; DMI, Desipramine; DOX, Doxepin; ESC, Escitalopram; FLX, Fluoxetine; FVX, Fluvoxamine; MAP, Maprotiline; NT, Nortriptyline; PAR, Paroxetine; VFX, Venlafaxine.

The combined effect estimate across 27 treatment arms from 19 studies included in the meta-analysis is −0.05 (−0.31, 0.21) [*p* ≤ 0.05, 95% confidence interval (CI), Hedges' g; Figure 1], which does not indicate a significant difference in antidepressant concentration between responders and non-responders. Information on the specific antidepressant concentrations, response criteria, and concentration determination are given in Supplementary Table 5.

Sensitivity analyses were mostly unremarkable. None of the analyses yielded significant results (Supplementary Table 6). Notably, none of the studies included in meta-analysis applied the MADRS for the assessment of depression severity, thus, only the HAMD was eligible for sensitivity analyses. However, exclusion of studies with mean concentrations outside of substance-specific therapeutic reference ranges greatly diminished heterogeneity.

### Identification of Quality Assessment Criteria as Moderators

**Figure 2:**
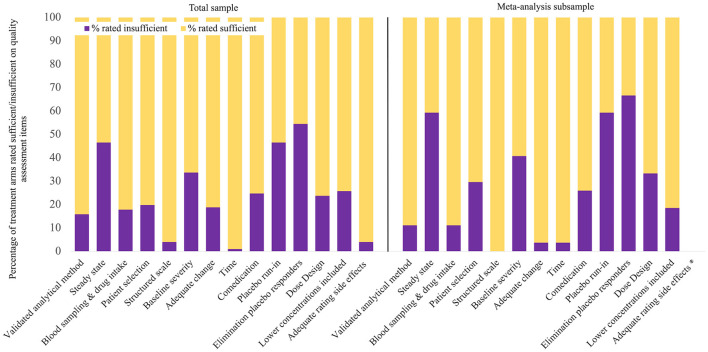
Quality assessment results. The total sample included 101 treatment arms from 65 studies, the meta-analysis subsample included 27 treatment arms from 19 studies, * Item 14 was only rated in studies investigating concentration-side effects associations, which was irrelevant in meta-analysis.

To investigate the potential of methodological properties of primary studies as moderators of the concentration-effect relationship, a quality assessment of all eligible studies (65 studies, including 101 treatment arms) on 14 criteria was conducted. The results are presented separately for all studies and the subsample of studies included in the meta-analysis in Figure 2. Criteria most often rated insufficient were *Elimination of placebo responders, Placebo run-in* and *Steady state*. This also applied for the meta-analysis subsample. On the other hand, *Structured scale, Time*, and *Adequate rating of side effects* were most often rated sufficient.

In qualitative synthesis, a meaningful interpretation of the impact of quality criteria as moderators of antidepressant concentration-effect relationships is restricted by the heterogeneity of findings across studies. Comparison of quality assessment ratings in the five decades for which publications were found indicated increasing quality until the 1990s, and a decline afterwards. Note that this association is observational and not based on statistical comparison. Results are reported in Supplementary Table 7.

A more systematic investigation of quality criteria as moderators of the concentration-efficacy relationship was possible in quantitative analysis, which was performed according to the three-step procedure described above. For this analysis, we report the following results.

I. Results from meta-regression including 12 eligible quality assessment criteria as dichotomous predictors (0 = insufficient, 1 = sufficient) revealed three significant or trend-level predictors: *Baseline severity* (*p* ≤ 0.05), *Dose design* (*p* ≤ 0.01), and *Lower concentrations included* (*p* ≤ 0.1). Detailed results are shown in Supplementary Figure 4. Positive numbers indicate higher effect sizes with a “sufficient” rating; this was the case for quality assessment criteria *Dose design* (estimate = 0.8762) and *Lower concentrations included* (estimate = 0.5812). Negative numbers signify smaller effect sizes with “sufficient” rating, as can be observed for *Baseline severity* (estimate = −0.7263). The total amount of heterogeneity accounted for by all predictors was estimated at *R*^2^ = 69.37%, which means that 69.37% of the differences in true effect sizes can be explained by the predictors included in the meta-regression.
II. To identify contributions of individual predictors, iterative ANOVAs were conducted with sequential inclusion of predictors. Results are displayed in Supplementary Figure 5. Significant or trend-level differences in model fit were observed for *Adequate change* and *Lower concentrations included* (*p* ≤ 0.1), *Time* (*p* ≤ 0.05), and *Dose design* (*p* ≤ 0.01). Including Akaike's information criterion with the correction for small sample sizes (AICc) as criterion of model superiority, the only favorable quality assessment criterion was *Dose design*, with a smaller AICc in the full than in the reduced model.
III. All potential moderators identified by either meta-regression or ANOVAs were validated by subgroup analyses, provided that there were at least three treatment arms per subgroup. Results are shown in Table 4. Two quality criteria yielded significant results in subgroup comparison, *Dose design* (Chi^2^ = 5.68, df = 1, *p* = 0.02, I^2^ = 82.4%) and *Lower concentrations included* (Chi^2^ = 3.74, df = 1, *p* = 0.05, I^2^ = 73.3%), indicating a significant moderating impact on the relationship between concentration and efficacy.

**Table 4:** Subgroup analyses of potential moderators.

**Criterion (*N* treatments arms)**	**Chi^**2**^**	**df**	***p***	***I* ^2^**
Baseline severity [Yes (16) vs. No (11)]	1.6	1	0.21	37.4%
Dose design [Fixed (18) vs. Flexible (9)]	5.68	1	0.02	82.4%
Lower concentrations included [Yes (20) vs. No (7)]	3.74	1	0.05	73.3%
**TCA subsample**				
Dose design [Fixed (12) vs. Flexible (4)]	6.09	1	0.01	83.6%
Lower concentrations included [Yes (12) vs. No (4)]	0.53	1	0.47	0%
**SSRI subsample**				
Dose design [Fixed (3) vs. Flexible (4)]	0.46	1	0.50	0%
Lower concentrations included [Yes (4) vs. No (3)]	3.87	1	0.05	74.2%

### Quality Assessment Criteria as Subgroup Variables

**Figure 3:**
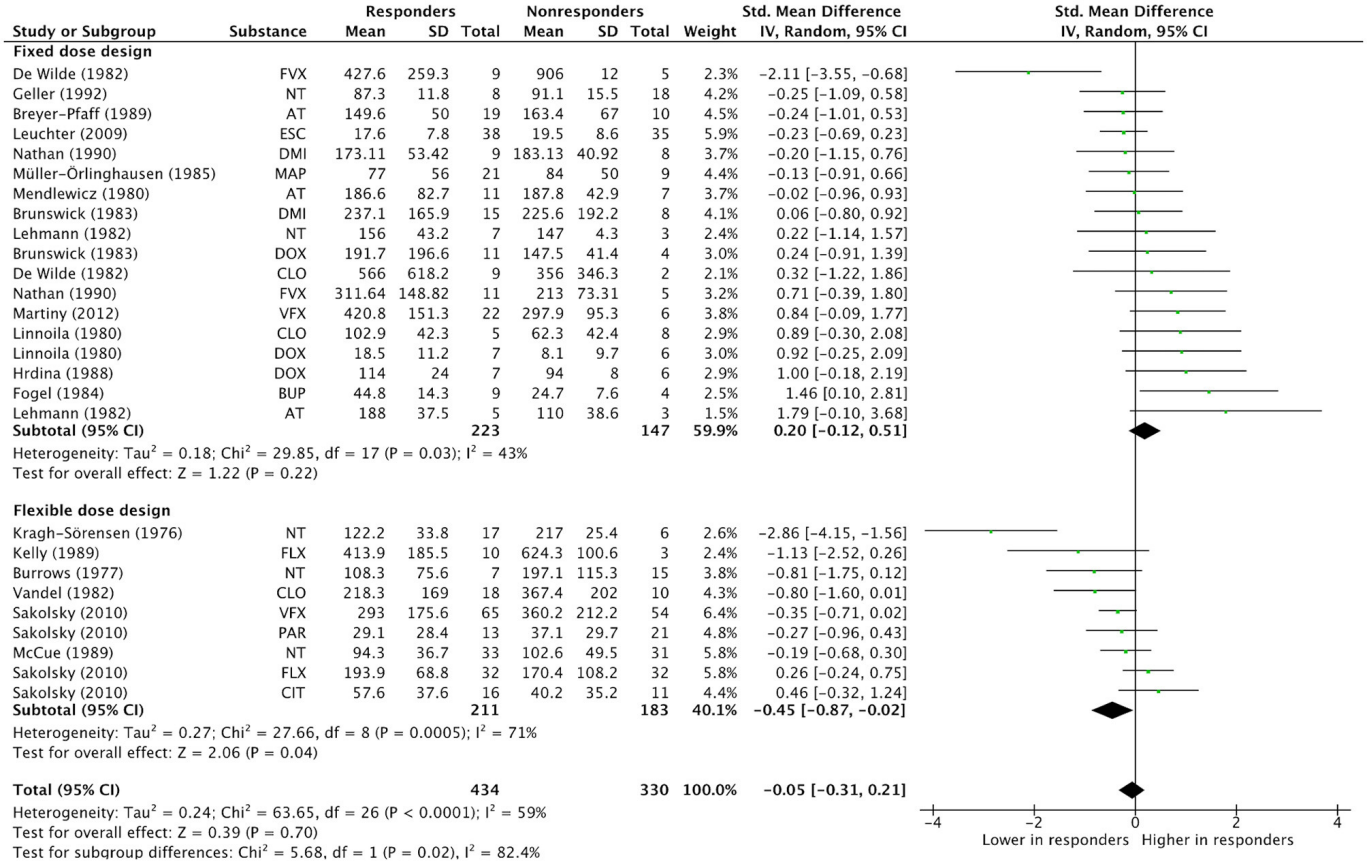
Subgroup analysis “Dose design”. Overall effect estimates across N treatment arms = 27, in *N* = 19 studies. AT, Amitriptyline; BUP, Bupropion; CIT, Citalopram; CLO, Clomipramine; DMI, Desipramine; DOX, Doxepin; ESC, Escitalopram; FLX, Fluoxetine; FVX, Fluvoxamine; MAP, Maprotiline; NT, Nortriptyline; PAR, Paroxetine; VFX, Venlafaxine.

**Figure 4:**
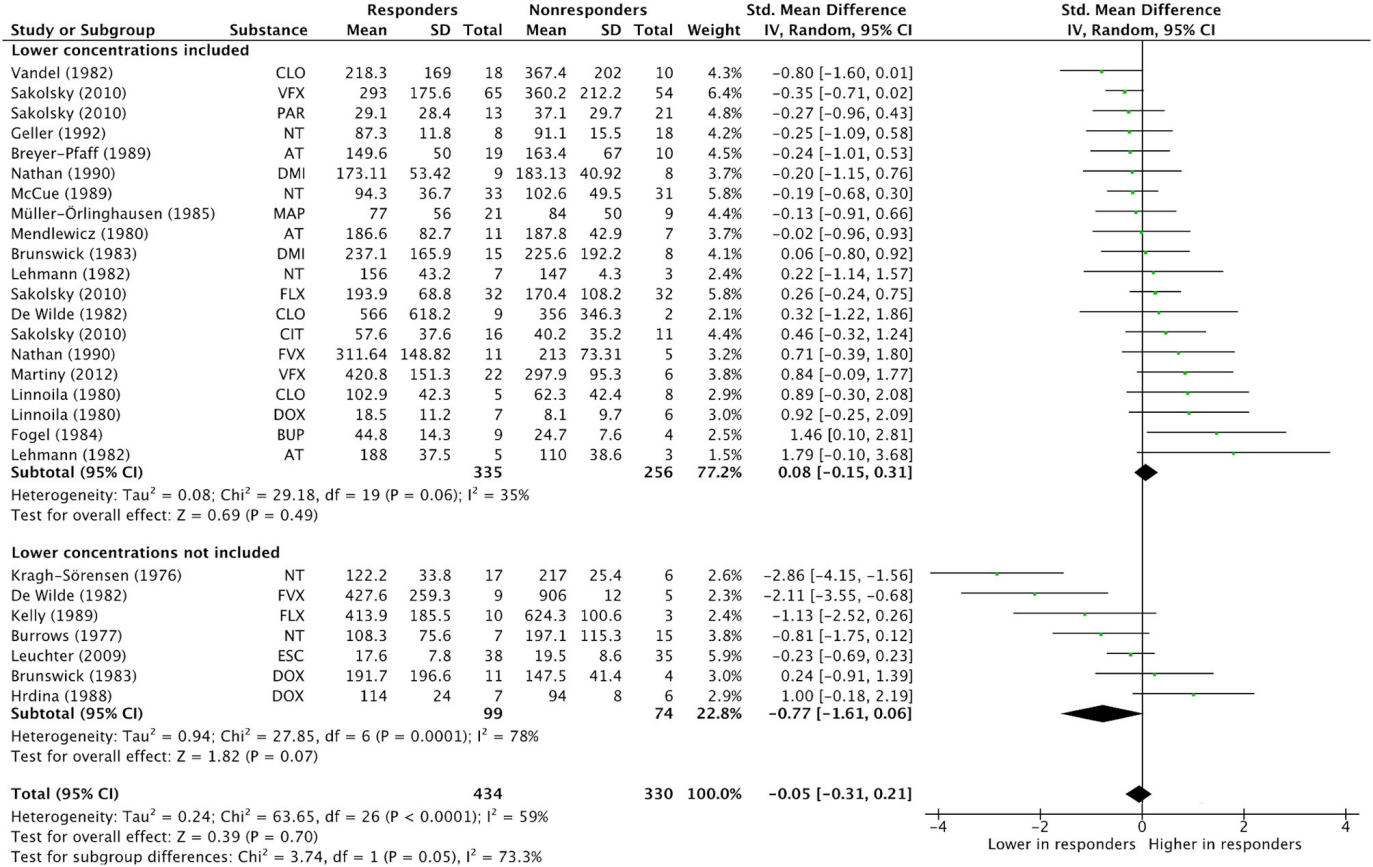
Subgroup analysis “Lower concentrations included”. Overall effect estimates across N treatment arms = 27, in *N* = 19 studies. AT, Amitriptyline; BUP, Bupropion; CIT, Citalopram; CLO, Clomipramine; DMI, Desipramine; DOX, Doxepin; ESC, Escitalopram; FLX, Fluoxetine; FVX, Fluvoxamine; MAP, Maprotiline; NT, Nortriptyline; PAR, Paroxetine; VFX, Venlafaxine.

To further investigate the impact of *Dose design* and *Lower concentrations included* as moderators, visual inspection of forest plots including subgroups of studies rated sufficient or insufficient are shown in Figures 3 and 4. The dosing protocol in primary studies moderates the relationship between concentration and efficacy significantly. The mean effect estimated across studies which used a fixed dose design was positive at 0.20 (CI −0.12, 0.51; *p* = 0.22, I^2^ = 43%), i.e., higher mean concentrations in responders than in non-responders, although this effect did not attain significance. The opposite pattern was observed in studies using a flexible dose design, with an overall subgroup effect estimate of −0.45 (CI −0.87, −0.02; *p* = 0.04, I^2^ = 71%), reflecting significantly lower concentrations in responders than in non-responders.

For quality assessment criterion *Lower concentrations included* as subgroup variable, a similar bidirectional effect size pattern is present: In studies including lower concentrations, the mean effect size was 0.08 (CI−0.15, 0.31; *p* = 0.49, I^2^ = 35%), whereas studies not including lower concentrations showed a mean effect size of −0.77 (CI −1.61, 0.06; *p* = 0.07, I^2^ = 78%). A significant concentration-efficacy relationship was not observed in either subgroup.

### TCA and SSRI Subsample Analyses

Separate subsample-analyses were conducted for the more homogenous groups of TCAs and SSRIs, including the previously identified significant moderators of effect (*Dose design* and *Lower concentrations included*). Results are displayed in Table 4. In the TCA subsample, including 16 treatment arms, *Dose design* significantly moderates the effect (Chi^2^ = 6.09, df = 1, *p* = 0.01, I^2^ = 83.6%), with mean effect sizes of 0.19 (−0.12, 0.49; *p* = 0.23, I^2^ = 0%) in the fixed dose subgroup and −1.03 (−1.94, −0.11; *p* = 0.03, I^2^ = 80%) in the flexible dose subgroup, reflecting significantly lower concentrations in responders than in non-responders. However, for *Lower concentrations included* as subgroup variable, subgroups were not significantly different (Chi^2^ = 0.53, df = 1, *p* = 0.47, I^2^ = 0%).

In the SSRI subsample, including seven treatment arms, no subgroup effect for the criterion *Dose design* was detected (Chi^2^ = 0.46, df = 1, *p* = 0.50, I^2^ = 0%). However, subgroup comparison is significant for criterion *Lower concentrations included* (Chi^2^ = 3.87, df = 1, *p* = 0.05, I^2^ = 74.2%), with treatment arms including lower concentrations showing an overall effect of 0.21 (−0.13, 0.56; *p* = 0.22, I^2^ = 1%), whereas treatment arms not including lower concentrations exhibited a negative overall effect of −1.00 (−2.17, 0.16; *p* = 0.09, I^2^ = 71%). A significant concentration-efficacy relationship could not be observed in either subgroup. However, the results were in line with the results and effect size pattern found in the analysis of the total sample.

### Further Subgroup Analyses

Additional subgroup analyses were performed to further explore sources of heterogeneity, investigating age of study participants, antidepressant classes, cumulative study quality, and publication date (before 1990 and after 1990) as subgroup variables. Results are shown in Supplementary Table 8. None of the subgroup comparisons attained significance.

## Discussion

In our study on the association between antidepressant concentrations and clinical effects in randomized controlled trials, we report the following results:

I. The majority of studies investigated TCAs (47 studies, 57 treatment arms), followed by SSRIs (21 studies, 27 treatment arms), tetracyclic antidepressants (9 studies, 9 treatment arms), SSNRIs (6 studies, 6 treatment arms), one MAO inhibitor (moclobemide; one study, one treatment arm) and one selective noradrenaline-dopamine reuptake inhibitor (bupropion; one study, one treatment arm).
II. The results from qualitative and overall quantitative synthesis generally reflect the current state of evidence on antidepressant concentration-effect research: Heterogenous findings and a high variability in quality and methods used in primary studies (23). In our meta-analysis, the heterogeneity is reflected by a wide range of effect estimates in single studies and a small overall effect. The majority of studies did not report findings of a concentration-efficacy relationship, and only a smaller fraction of studies investigated concentration-side effects associations. The assessment of side effects was highly heterogenous. Valid conclusions could not be drawn from these results. Assessment of treatment efficacy was more homogenous. Notably, historical changes of diagnostic criteria and rating instruments for depressive disorders are a source of between-study heterogeneity.
III. However, the results from this study provide statistical evidence for the impact of moderating factors, which substantially influence the relationship between antidepressant concentration and efficacy. The investigation of such sources of heterogeneity and their specific impact on concentration-efficacy associations was the main goal of this study. Due to the influence of these moderators, the currently published research collectively does not allow a reliable inference on the relationship between TDM and clinical outcomes.

Although impact of such moderating factors has been hypothesized by multiple authors (9, 11, 22, 23, 41–44), statistical support for an influence of these moderators on the association between antidepressant concentration and clinical efficacy has so far not been provided. By performing exploratory analyses (meta-regression and ANOVAs) of the impact of previously described potential moderators of concentration-efficacy relationships, we were able to identify two methodological properties of primary studies which significantly moderate the association between antidepressant concentration and efficacy:

First, we could demonstrate a substantial impact of the dose design used in primary studies on the association between antidepressant concentration and efficacy. The use of a fixed dose design has already been described as the best way to address the question whether there is a relationship between concentration and effect, not only in antidepressant treatment, but in pharmacological treatment in general (32, 41). Flexible dose studies are often designed to provide evidence for antidepressant efficacy in comparison to placebo or active control groups. By adjusting doses according to non-response or development of side effects, the efficacy of antidepressant drugs may be enhanced in comparison to control groups in these studies. However, a meaningful relationship between concentration and efficacy may be obscured by the flexible study designs: It has been theorized that inverse relationships between concentration and efficacy could result from higher dosing in non-responders and lowered dosing in case of side effects in studies employing a flexible dose design (45). The opposite direction of effects in fixed and flexible dose design studies in our subgroup analysis provides strong support for this hypothesis.

Second, another significant moderator of the relationship between concentration and efficacy was the inclusion or exclusion of antidepressant concentrations in the lower- or subtherapeutic range in primary studies. As has been described recently (22), neuropsychopharmacological drugs tend to exhibit two opposite directions of effect across their respective range of concentrations. In lower concentration ranges, there seems to be a positive direction of effect, increasing efficacy with increasing concentrations. However, in higher concentration ranges, a negative direction could be observed, indicating a decline in efficacy with increasing concentrations. This biphasic concentration-efficacy relationship has already been shown for TCAs in systematic reviews (11, 12) and in a recent mega-analysis including four newer antidepressants (mirtazapine, escitalopram, duloxetine, and venlafaxine) (46).

In our analysis, the higher concentrations in non-responders than in responders in studies not including lower concentrations might therefore be caused by a deterioration of efficacy or a higher degree of side effects, potentially confounding efficacy assessments in higher concentration ranges.

Several limitations of this study have to be addressed: A general limitation is the higher number of studies investigating TCAs, both in the qualitative review and the meta-analysis. However, considering the weight of studies, which directly influences their impact on effect estimates, non-TCA treatment arms make up for 45.7% of the total weight of studies included in the meta-analysis.

Notably, results from separate analyses in TCA and SSRI subsamples showed that for TCAs, *Dose design* significantly moderates the association between concentration and efficacy, with higher concentrations in non-responders than in responders in flexible dose studies. *Lower concentrations included* did not significantly moderate the effect in the TCA subsample. However, in the SSRI subsample, the inclusion or exclusion of lower concentrations significantly moderated the association between antidepressant concentration and efficacy, with bidirectional effect size patterns, a positive subgroup effect estimate in studies including lower concentrations, and a negative subgroup effect estimate in studies not including lower concentrations. *Dose design* did not significantly moderate the association between concentration and efficacy in the SSRI subsample. These results must be interpreted with regard to the smaller number of available treatment arms, which may have limited the power of the analyses.

Other sources of between-study heterogeneity might also have implications for the interpretation of overall results. The present study included results from adolescents, adults, and older adults. Subgroup analysis investigating a potential impact of age did not yield significant results, although the number of studies with older adult participants was too small for inclusion in subgroup comparison. As mentioned before, it became apparent that efficacy and side effect reports differed substantially in primary studies, which must be considered in the interpretation of combined results. Furthermore, the methods used for investigating concentration-efficacy relationships differed greatly. While some studies reported correlations between concentration and efficacy, others used concentration values as predictors of treatment response. Comparisons of concentrations in treatment responders and non-responders, as well as numbers of responders in predefined concentration ranges were used as outcome measurements, too. This diversity limits the interpretation of results from qualitative synthesis. Additionally, some studies reported concentration-effect relationships for a combination of treatment arms whenever the results were in the same direction. While this does not have implications for the direction of effect, differences in the magnitude of concentration-effect relationships were not considered. Finally, one major reason for between-study heterogeneity were differences in diagnoses as well as classification systems. In quantitative synthesis, this could be accounted for by sensitivity analyses investigating the influence of differences in depression rating systems and classifications. None of the analyses showed significant results. Furthermore, it has been argued that ratings on scales frequently used in the assessment of depression may be regarded as equivalent (47). Finally, another potential source of between-study heterogeneity might have been the broad range of publication dates of studies included in qualitative and quantitative analysis, with the earliest studies published in the 1970s. As shown in Supplementary Table 7, overall quality assessment increased until the 1990s, however, it declined afterwards. In an attempt to quantitatively investigate the potential impact of publication date, a subgroup analysis of studies published before and after 1990 did not exhibit significant results.

Limitations concerning the quality assessment of studies pertain to several aspects: Treatment arms were rated insufficient whenever information on the quality assessment criterion was missing. This approach might have led to underestimating the quality of some studies. Moreover, it was necessary to define operationalizations for criteria which applied to all substances. However, this general approach might not have accounted for the variability between substances. For example, quality assessment criterion *Lower concentrations included* was rated sufficient if an interval of 10% around the lowest defined therapeutic concentration (9) was met. However, for substances with a narrower therapeutic range, a small difference in concentration might be associated with a greater difference in effect than it would for drugs with a wider therapeutic range and a lower toxicity.

Another relevant aspect was the assessment and report of active metabolites in primary studies, which was not always the case. However, the active metabolites have an influence on treatment effect and thus on the relationship between concentration and effect. Notably, none of the studies included in quantitative synthesis failed to report data for active metabolites.

Finally, due to the study design, we only investigated quality criteria which were identified as potential moderators *via* meta-regression or ANOVAs. This led to disregarding other quality assessment criteria, which did not yield at least trend level results in the described analyses for the final subgroup analysis. However, many of the proposed quality criteria were too homogenous across studies to be able to determine meaningful subgroup differences between studies rated “sufficient” or “insufficient” on the respective criteria.

To address the titular question of the present study, whether TDM is relevant in antidepressant drug treatment, we provided statistical evidence that the fundamental precondition for relevance of TDM in antidepressant treatment, a relationship between concentration and effect, cannot be answered with certainty based on the randomized-controlled trials available today. As has been demonstrated, methodological deficits of primary studies may have prevented past research from finding significant associations between the variables, thus underestimating the relevance of TDM in enabling a more efficient and safer antidepressant drug treatment.

## Conclusion

The present study is the first study to date to provide statistical evidence for the impact of methodological shortcomings in primary studies on the relationship between antidepressant concentration and efficacy. These methodological shortcomings may have prevented past research on antidepressants from finding evidence for the basic assumption of TDM, association between concentration and efficacy. Future research should consider these results in the design of studies investigating concentration-efficacy relationships or use the applied method for investigating moderators in other drug classes. Since evidence for concentration effect relationship is poor not only for antidepressant drugs, it may be assumed that the observed shortcomings are also relevant for other psychotropic and non-psychotropic drugs.

## Data Availability Statement

The original contributions presented in the study are included in the article/Supplementary Material, further inquiries can be directed to the corresponding author/s.

## Author Contributions

CSMF and TGR conceptualized the study, acquired, curated, analyzed the data, and collaborated on the original draft. XMH assisted in the quality assessment and performed supporting literature research. XMH, GG, CH, BE, and RK provided input on the study design and participated in the interpretation of the data. RK additionally provided research resources. All authors participated in reviewing the manuscript. All authors contributed to the article and approved the submitted version.

## Conflict of Interest

GG has served as a consultant for Allergan, Boehringer Ingelheim, Institute for Quality and Efficiency in Health Care (IQWiG), Janssen-Cilag, Lundbeck, Otsuka, Recordati, ROVI, Sage, and Takeda. He has served on the speakers' bureau of Gedeon Richter, Janssen Cilag, Lundbeck, Otsuka, Recordati. He has received grant support from Boehringer Ingelheim, Lundbeck and Saladax. He is co-founder and/or shareholder of Mind and Brain Institute GmbH, Brainfoods GmbH, OVID Health Systems GmbH and MIND Foundation gGmbH. RK reports modest honoraria for consultancy, lectures, and support for research from Bayer Pharma, Berlin-Chemie Menarini, Daiichi Sankyo, Ferrer, Sanofi, and Servier outside the submitted work.

The remaining authors declare that the research was conducted in the absence of any commercial or financial relationships that could be construed as a potential conflict of interest.

## Publisher's Note

All claims expressed in this article are solely those of the authors and do not necessarily represent those of their affiliated organizations, or those of the publisher, the editors and the reviewers. Any product that may be evaluated in this article, or claim that may be made by its manufacturer, is not guaranteed or endorsed by the publisher.

## References

[1] Vos T Lim SS Abbafati C Abbas KM Abbasi M Abbasifard M . Global burden of 369 diseases and injuries in 204 countries and territories, 1990–2019: a systematic analysis for the Global Burden of Disease Study 2019. Lancet. (2020) 396:1204–22. 10.1016/S0140-6736(20)30925-9 33069326 PMC7567026

[2] DGPPN BÄK KBV AWMF (Hrsg.) für die Leitliniengruppe Unipolare Depression^*^. S3-Leitlinie/Nationale Versor- gungsLeitlinie Unipolare Depression – Langfassung, 2. Auflage. Version 5 (2015). Available online at: www.depression.versorgungsleitlinien.de (accessed October 1, 2022).

[3] Cipriani A Furukawa TA Salanti G Chaimani A Atkinson LZ Ogawa Y . Comparative efficacy and acceptability of 21 antidepressant drugs for the acute treatment of adults with major depressive disorder: a systematic review and network meta-analysis. Lancet. (2018) 391:1357–66. 10.1016/S0140-6736(17)32802-7 29477251 PMC5889788

[4] Hiemke C. Clinical utility of drug measurement and pharmacokinetics: therapeutic drug monitoring in psychiatry. Eur J Clin Pharmacol. (2008) 64:159–66. 10.1007/s00228-007-0430-1 18196227

[5] von Knorring L Akerblad AC Bengtsson F Carlsson A Ekselius L. Cost of depression: effect of adherence and treatment response. Eur Psychiatry. (2006) 21:349–54. 10.1016/j.eurpsy.2006.04.005 16777385

[6] Lloret-Linares C Bellivier F Haffen E Aubry JM Daali Y Heron K . Markers of individual drug metabolism: towards the development of a personalized antidepressant prescription. Curr Drug Metab. (2015) 16:17–45. 10.2174/138920021601150702160728 26152128

[7] Grundmann M Kacirova I Urinovska R. Therapeutic monitoring of psychoactive drugs - antidepressants: a review. Biomed Pap Med Fac Univ Palacky Olomouc Czech Repub. (2015) 159:35–43. 10.5507/bp.2013.020 23549513

[8] Clarke W. Chapter 1 - Overview of therapeutic drug monitoring. In: Clarke W Dasgupta A editors. Clinical Challenges in Therapeutic Drug Monitoring. San Diego: Elsevier (2016). p. 1–15. 10.1016/B978-0-12-802025-8.00001-5

[9] Hiemke C Bergemann N Clement HW Conca A Deckert J Domschke K . Consensus guidelines for therapeutic drug monitoring in neuropsychopharmacology: update 2017. Pharmacopsychiatry. (2018) 51:e1. 10.1055/s-0037-1600991 29390205

[10] Aronson JK Hardman M. ABC of monitoring drug therapy. Measuring plasma drug concentrations. BMJ. (1992) 305:1078–80. 10.1136/bmj.305.6861.1078 1467691 PMC1883634

[11] Ulrich S Läuter J. Comprehensive survey of the relationship between serum concentration and therapeutic effect of amitriptyline in depression. Clin Pharmacokinet. (2002) 41:853–76. 10.2165/00003088-200241110-00004 12190332

[12] Perry PJ Zeilmann C Arndt S. Tricyclic antidepressant concentrations in plasma: an estimate of their sensitivity and specificity as a predictor of response. J Clin Psychopharmacol. (1994) 14:230–40. 10.1097/00004714-199408000-00002 7962678

[13] Lohse MJ. Psychopharmaka. In: Schwabe U Ludwig W-D editors. Arzneiverordnungs-Report 2020. Berlin, Heidelberg: Springer Berlin Heidelberg (2020). p. 781–814.

[14] Mitchell PB. Therapeutic drug monitoring of non-tricyclic antidepressant drugs. Clin Chem Lab Med. (2004) 42:1212–8. 10.1515/CCLM.2004.243 15576285

[15] Tricyclic antidepressants–blood level measurements and clinical outcome: an APA Task Force report. Task Force on the use of laboratory tests in psychiatry. Am J Psychiatry. (1985) 142:155–62. 10.1176/ajp.142.2.155 3881999

[16] Preskorn SH Burke MJ Fast GA. Therapeutic drug monitoring. Principles and practice. Psychiatr Clin North Am. (1993) 16:611–45. 10.1016/S0193-953X(18)30167-9 8415237

[17] Balant-Gorgia E Balant L. Therapeutic drug monitoring: relevance during the drug treatment of psychiatric disorders. Drug Ther CNS Drug. (1995) 4:432–53. 10.2165/00023210-199504060-00006

[18] Burke MJ Preskorn SH. Therapeutic drug monitoring of antidepressants: cost implications and relevance to clinical practice. Clin Pharmacokinet. (1999) 37:147–65. 10.2165/00003088-199937020-00004 10496302

[19] Rasmussen BB Brosen K. Is therapeutic drug monitoring a case for optimizing clinical outcome and avoiding interactions of the selective serotonin reuptake inhibitors? Ther Drug Monit. (2000) 22:143–54. 10.1097/00007691-200004000-00001 10774624

[20] Wille SM Cooreman SG Neels HM Lambert WE. Relevant issues in the monitoring and the toxicology of antidepressants. Crit Rev Clin Lab Sci. (2008) 45:25–89. 10.1080/10408360701713112 18293180

[21] Hefner G Laib AK Sigurdsson H Hohner M Hiemke C. The value of drug and metabolite concentration in blood as a biomarker of psychopharmacological therapy. Int Rev Psychiatry. (2013) 25:494–508. 10.3109/09540261.2013.836475 24151798

[22] Zernig G Hiemke C. Pharmacokinetic and Pharmacodynamic Principles. In: Riederer P Laux G Nagatsu T Le W Riederer C editors. NeuroPsychopharmacotherapy. Cham: Springer International Publishing (2020). p. 1–19.

[23] Kloosterboer SM Vierhout D Stojanova J Egberts KM Gerlach M Dieleman GC . Psychotropic drug concentrations and clinical outcomes in children and adolescents: a systematic review. Expert Opin Drug Saf. (2020) 19:873–90. 10.1080/14740338.2020.1770224 32421365

[24] Ulrich S Wurthmann C Brosz M Meyer FP. The relationship between serum concentration and therapeutic effect of haloperidol in patients with acute schizophrenia. Clin Pharmacokinet. (1998) 34:227–63. 10.2165/00003088-199834030-00005 9533984

[25] Page MJ McKenzie JE Bossuyt PM Boutron I Hoffmann TC Mulrow CD . The PRISMA 2020 statement: an updated guideline for reporting systematic reviews. Syst Rev. (2021) 10:89. 10.1186/s13643-021-01626-4 33781348 PMC8008539

[26] Agoritsas T Merglen A Courvoisier DS Combescure C Garin N Perrier A . Sensitivity and predictive value of 15 PubMed search strategies to answer clinical questions rated against full systematic reviews. J Med Internet Res. (2012) 14:e85. 10.2196/jmir.2021 22693047 PMC3414859

[27] Sterne JAC Savovic J Page MJ Elbers RG Blencowe NS Boutron I . RoB 2: a revised tool for assessing risk of bias in randomised trials. BMJ. (2019) 366:l4898. 10.1136/bmj.l4898 31462531

[28] McGuinness LA Higgins JPT. Risk-of-bias VISualization (robvis): an R package and Shiny web app for visualizing risk-of-bias assessments. Res Synth Methods. (2020) 12:55–61. 10.1002/jrsm.1411 32336025

[29] Egger M Davey Smith G Schneider M Minder C. Bias in meta-analysis detected by a simple, graphical test. BMJ. (1997) 315:629–34. 10.1136/bmj.315.7109.629 9310563 PMC2127453

[30] R Core Team. R: A Language Environment for Statistical Computing. Vienna, Austria: R Foundation for Statistical Computing (2021). Available online at: https://www.R-project.org/ (accessed November 21, 2021).

[31] Balduzzi S Rucker G Schwarzer G. How to perform a meta-analysis with R: a practical tutorial. Evid Based Ment Health. (2019) 22:153–60. 10.1136/ebmental-2019-300117 31563865 PMC10231495

[32] Hart XM Eichentopf L Lense X Riemer T Wesner K Hiemke C . Therapeutic reference ranges for psychotropic drugs: a protocol for systematic reviews. Front Psychiatry. (2021) 12:787043. 10.3389/fpsyt.2021.787043 34899439 PMC8653700

[33] The Cochrane Collaboration. Review Manager (RevMan) [Computer program]. Version 5.4 ed (2020). Available online at: https://training.cochrane.org/online-learning/core-software-cochrane-reviews/revman/revman-5-download (accessed November 21, 2021).

[34] Hamilton M. A rating scale for depression. J Neurol Neurosurg Psychiatry. (1960) 23:56–62. 10.1136/jnnp.23.1.56 14399272 PMC495331

[35] Montgomery SA Asberg M. A new depression scale designed to be sensitive to change. Br J Psychiatry. (1979) 134:382–9. 10.1192/bjp.134.4.382 444788

[36] Harrer M Cuijpers P Furukawa TA Ebert DD. Doing Meta-Analysis With R: A Hands-On Guide. 1st ed. Boca Raton, FL London: Chapman & Hall/CRC Press (2021). Available online at: https://bookdown.org/MathiasHarrer/Doing_Meta_Analysis_in_R/ (accessed November 21, 2021).

[37] Viechtbauer W. Conducting meta-analyses in R with the metafor package. J Stat Software. (2010) 36:1–48. 10.18637/jss.v036.i03

[38] Klotz U. Pharmacokinetics and drug metabolism in the elderly. Drug Metab Rev. (2009) 41:67–76. 10.1080/03602530902722679 19514965

[39] Egberts KM Mehler-Wex C Gerlach M. Therapeutic drug monitoring in child and adolescent psychiatry. Pharmacopsychiatry. (2011) 44:249–53. 10.1055/s-0031-1286291 21959786

[40] Furukawa TA Cipriani A Leucht S Atkinson LZ Ogawa Y Takeshima N . Is placebo response in antidepressant trials rising or not? A reanalysis of datasets to conclude this long-lasting controversy. Evid Based Ment Health. (2018) 21:1–3. 10.1136/eb-2017-102827 29330216 PMC10270408

[41] Hiemke C. Concentration-effect relationships of psychoactive drugs and the problem to calculate therapeutic reference ranges. Ther Drug Monit. (2019) 41:174–9. 10.1097/FTD.0000000000000582 30883511

[42] Laux G Baumann P Hiemke C. Therapeutic drug monitoring of antidepressants — clinical aspects. In: Gerlach M Deckert J Double K Koutsilieri E editors. Neuropsychiatric Disorders - An Integrative Approach. Vienna: Springer Vienna (2007). p. 261–86. Available online at: https://link.springer.com/book/10.1007/978-3-211-73574-9?page=2#toc (accessed November 21, 2021).

[43] Baumann P Hiemke C Ulrich S Eckermann G Gaertner I Gerlach M . The AGNP-TDM expert group consensus guidelines: therapeutic drug monitoring in psychiatry. Pharmacopsychiatry. (2004) 37:243–65. 10.1055/s-2004-832687 15551191

[44] Bengtsson F. Therapeutic drug monitoring of psychotropic drugs. TDM “nouveau”. Ther Drug Monit. (2004) 26:145–51. 10.1097/00007691-200404000-00010 15228155

[45] Preskorn SH. Therapeutic drug monitoring (TDM) in psychiatry (part I): why studies attempting to correlate drug concentration and antidepressant response don't work. J Psychiatr Pract. (2014) 20:133–7. 10.1097/01.pra.0000445247.54048.68 24638047

[46] Cellini L De Donatis D Zernig G De Ronchi D Giancarlo G Serretti A . Antidepressant efficacy is correlated with plasma levels: mega analysis and further evidence. Int Clin Psychopharmacol. (2021) 37:29–37. 10.1097/YIC.0000000000000386 34908537 PMC9648983

[47] Riedel M Moller HJ Obermeier M Schennach-Wolff R Bauer M Adli M . Response and remission criteria in major depression–a validation of current practice. J Psychiatr Res. (2010) 44:1063–8. 10.1016/j.jpsychires.2010.03.006 20447651

