# Supplementary material for: Is Therapeutic Drug Monitoring Relevant for Antidepressant Drug Therapy? Implications From a Systematic Review and Meta-Analysis With Focus on Moderating Factors
Source: Front Psychiatry. 2022 Feb 21;13:826138. doi: 10.3389/fpsyt.2022.826138 (PMC8898907; doi:10.3389/fpsyt.2022.826138)
Supplement: Supplementary file 1 [file Data_Sheet_1.docx]

Supplementary Material

**Tables**

| Table S1. Summary of Reviews from 1977 to 2021 | | |
| --- | --- | --- |
| Review | **Title** | **Conclusion** |
| Zernig and Hiemke (2020) (1) | Pharmacokinetic and Pharmacodynamic Principles | The authors review evidence on concentration-effect relationships in pharmacological drugs, which is described as generally heterogenous. Reasons for this lack of evidence are reviewed systematically and summarized into a study design checklist, which, if followed, might provide a more valid investigation of the true relationship between concentration and effect. |
| Kloosterboer et al. (2020) (2) | Psychotropic Drug Concentrations and Clinical Outcomes in Children and Adolescents: A Systematic Review | This review reports relationships between concentrations and clinical outcomes found in single studies. The authors note that these results lack replication. Future research should consider methodological properties of primary studies, which may obscure concentration-effect associations. |
| Protti et al. (2020) (3) | New-generation, Non-SSRI Antidepressants: Drug-drug Interactions and Therapeutic Drug Monitoring. Part 2: NaSSAs, NRIs, SNDRIs, MASSAs, NDRIs, and Others | Personalization of antidepressant therapy is needed to enhance treatment efficacy. Data on chemical-clinical correlations for newer antidepressants and the development of valid analytical methods should be produced to make TDM a useful tool, also for newer antidepressant agents. |
| Hiemke (2019) (4) | Concentration-Effect Relationships of Psychoactive Drugs and the Problem to Calculate Therapeutic Reference Ranges | Findings on psychoactive drug concentration-effect relationships depend strongly on study design. Flexible dose studies cannot provide information about possible concentration-effect correlations. |
| Hiemke et al. (2018) (5) | Consensus Guidelines for Therapeutic Drug Monitoring in Neuropsychopharmacology: Update 2017 | Evidence for a concentration-effect relationship and therapeutic reference ranges has been provided over the past decades for antidepressant drugs, mostly tricyclic antidepressants. A general methodological problem, which obscures findings of such a relationship in studies, is described as the use of a flexible-dose design. |
| Mandrioli et al. (2018) (6) | New-Generation, Non-SSRI Antidepressants: Therapeutic Drug Monitoring and Pharmacological Interactions. Part 1: SNRIs, SMSs, SARIs | The authors highlight the importance of personalization in antidepressant therapy and underline the usefulness of TDM. Applying TDM in newer antidepressant drugs requires the development of validated analytical methods. |
| Grundmann et al. (2015) (7) | Therapeutic Monitoring of Psychoactive Drugs – Antidepressants: A Review | Monitoring of drug concentration is necessary due to a high interindividuality in pharmacokinetic variability of antidepressant concentration in blood. TDM enables individualization of antidepressant treatment and can enhance treatment efficacy and safety. |
| Lloret-Linares et al. (2015) (8) | Markers of Individual Drug Metabolism: Towards the Development of a Personalized Antidepressant Prescription | Identification of individual markers determining variability in drug metabolism may improve antidepressant therapy by leading to more personalized treatment.  Regarding concentration-effect relationships for antidepressants, the authors describe results from studies showing favorable and less favorable clinical outcomes above/below set concentration cut-offs for TCAs, citalopram, and other SSRIs.  *continued* |
| Hefner et al. (2013) (9) | The Value of Drug and Metabolite Concentration in Blood as Biomarker of Psychopharmacological Therapy. | Drug and metabolite concentrations of psychopharmacological agents can be used as biomarkers in antidepressant drug treatment.TDM is described as a useful tool to enhance efficacy and safety of pharmacotherapy, particularly in elderly patient  populations. Evidence on the relationship between drug concentration and clinical efficacy and side effects is reviewed. For TCAs, multiple studies suggesting an association of concentration and clinical efficacy are summarized, while the situation is described as less clear for newer antidepressant agents, mostly due to lack of suitable studies. For side effects, evidence on the association between TCA concentration and seizures, cardiac effects, and anticholinergic effects is presented. For SSRIs, a lack of studies on the relationship between concentration and side effects is described. |
| Mandrioli et al. (2012) (10) | Selective Serotonin Reuptake Inhibitors (SSRIs): Therapeutic Drug Monitoring and Pharmacological Interactions | The review describes the main pharmacodynamic and pharmacokinetic characteristics of six SSRIs. SSRI treatment, while useful and safe in antidepressant treatment, can benefit greatly from TDM to enhance efficacy and safety. |
| Gründer et al. (2011) (11) | Therapeutic Plasma Concentrations of Antidepressants and Antipsychotics: Lessons from PET Imaging | Therapeutic reference ranges necessary for the successful implementation of TDM in patient treatment can be validated successfully by investigating the relationship between brain target occupancy measured via PET and blood concentration of psychotropic drugs. |
| Pichini et al. (2009) (12) | Pharmacokinetics and Therapeutic Drug Monitoring of Psychotropic Drugs in Pediatrics | Pharmacokinetic parameters of psychotropic drugs in pediatric populations are reviewed, the specific importance of TDM in the pediatric setting is underlined as well as the need for further studies investigating pharmacokinetic properties of psychotropic drugs in children. |
| Hiemke (2008) (13) | Therapeutic drug monitoring in neuropsychopharmacology: does it hold its promise? | The review highlights the potential benefits of TDM use in psychopharmacological treatment and the need for additional studies investigating the relationship between drug concentration and clinical effects. |
| Wille et al. (2008) (14) | Relevant Issues in the Monitoring and the Toxicology of Antidepressants | Fifteen antidepressant agents are reviewed with respect to their specific indication for TDM use. Pharmacological properties as well as clinical effects are described. The review gives differential recommendations for TDM, mainly to enhance safety in treatment with older antidepressants and to improve treatment with new-generation antidepressants. The authors point out that a concentration-effect relationship could not be deduced clearly for newer antidepressants. |
| Hiemke (2008) (15) | Clinical Utility of Drug Measurement and Pharmacokinetics – Therapeutic Drug Monitoring in Psychiatry | The review highlights benefits of TDM use in clinical practice as well as problems in the implementation of the technique in routine care. It also emphasizes the need for future high-quality studies investigating the relationship between psychopharmacological drug concentrations and clinical effects. |
| Laux et al. (2007) (16) | Therapeutic Drug Monitoring of Antidepressants – Clinical Aspects | The relationship between plasma concentrations and clinical variables is described as the basic assumption of TDM. Evidence on the association between the variables is described as inconsistent. The authors argue that this deficit is most likely due to methodological shortcomings in studies investigating this relationship.  They refer to systematic reviews and meta-analyses which included methodologically suitable studies for their analyses and were able to demonstrate concentration-effect  relationships for nortriptyline, imipramine, desipramine, and amitriptyline.  *continued* |
| DeVane et al. (2006) (17) | Therapeutic Drug Monitoring of Psychoactive Drugs During Pregnancy in the Genomic Era: Challenges and Opportunities | The specific importance of pharmacokinetic studies in pregnant women treated with psychopharmacological drugs is highlighted. TDM is described as a particularly useful tool to enhance treatment safety in this specific patient population. |
| Hendset et al. (2006) (18) | The Complexity of Active Metabolites in Therapeutic Drug Monitoring of Psychotropic Drugs | This review describes the impact of (active) metabolites in psychopharmacological treatment and the specific problem of different pharmacological potential of drug and metabolite. Considering weighted plasma concentrations rather than the sum of parent drug and metabolite might increase the value of TDM. |
| Eap et al. (2004) (19) | Therapeutic Monitoring of Antidepressants in the Era of Pharmacogenetic Studies | The potential of pharmacogenetics and TDM in antidepressant treatment is reviewed. Both methods can act complementary in improving treatment of depressive disorders. |
| Bengtsson (2004) (20) | Therapeutic Drug Monitoring of Psychotropic Drugs – TDM „nouveau“ | The author proposes an extension to previous TDM methods by including within-person concentration measurements as reference points for further dose adjustments instead of population-based approaches. The author shortly describes the lack of evidence on concentration-effect relationships in psychotropic drugs, concluding that this lack might largely be due to methodological deficits in studies investigating this association. |
| Mitchell (2004) (21) | Therapeutic Drug Monitoring of Non-tricyclic Antidepressant Drugs | Non-TCA antidepressants and their potential indication for TDM are reviewed. The author concludes that a relationship between antidepressant concentration and clinical effects could not be demonstrated. The author sees utility of TDM in newer antidepressants in case of nonresponse to adequate treatment. |
| Baumann et al. (2004) (22) | The AGNP-TDM Expert Group Consensus Guidelines: Therapeutic Drug Monitoring in Psychiatry | A relationship between drug concentration and clinical effects is described as the basic assumption of TDM. Heterogeneity in findings on the relationship is attributed by the authors to methodological deficits in primary studies investigating the association. Reviews including studies with adequate design, however, were able to demonstrate such a relationship for nortriptyline, imipramine, and desipramine for antidepressant agents. |
| Ulrich and Läuter (2002) (23) | Comprehensive Survey of the Relationship Between Serum Concentration and Therapeutic Effect of Amitriptyline in Depression | Evidence on a concentration-therapeutic effect relationship in antidepressant amitriptyline treatment is reviewed. The authors conclude a biphasic relationship and point out the need for further controlled and randomized studies on the matter. |
| Mitchell (2001) (24) | Therapeutic Drug Monitoring of Psychotropic Medication | The use of TDM in psychiatry is reviewed. Regarding antidepressants, the author highlights the usefulness of TDM in TCAs, but does not extend this conclusion to newer antidepressants both for lack of evidence of a therapeutic reference range and due to lower toxicity. |
| Rasmussen and Brösen (2000) (25) | Is Therapeutic Drug Monitoring a Case for Optimizing Clinical Outcome and Avoiding Interactions of the Selective Serotonin Reuptake Inhibitors? | Evidence on a potential relationship between clinical efficacy and plasma concentrations in SSRIs is reviewed. There does not seem to be a meaningful relationship, which limits the usefulness of TDM in SSRI treatment.  It may, however, be useful in the monitoring of compliance, in case of side effects, as well as in elderly patients and patients with liver impairment. |
| Burke and Preskorn (1999) (26) | Therapeutic Drug Monitoring of Antidepressants – Cost Implications and Relevance to Clinical Practice | The authors review different classes of antidepressant agents with respect to pharmacological features and recommendations for TDM.  TDM is recommended for TCAs due a narrow therapeutic range and for SSRIs, as there is some evidence on a concentration-clinical effects relationship.  Other classes of antidepressants which might profit from TDM are discussed without a clear conclusion. *continued* |
| Balant-Gorgia and Balant (1995) (27) | Therapeutic Drug Monitoring – Relevance During the Drug Treatment of Psychiatric Disorders | The authors review evidence for the usefulness of TDM in psychiatric care. A relationship between concentration and response in TCAs is described. |
| Preskorn et al. (1993) (28) | Therapeutic Drug Monitoring – Principles and Practices | Evidence on the usefulness of TDM and concentration-effect relationships in psychopharmacological treatment is reviewed. Among antidepressants, TCAs are the only agents for which routine TDM application is recommended. For SSRIs, the lack of a concentration-effect relationship is described, and TDM is recommended in case of unclear compliance or possible drug-drug interactions. |
| Perry et al. (1987) (29) | The Relationship Between Antidepressant Response and Tricyclic Antidepressant Plasma Concentrations – A Retrospective Analysis of the Literature Using Logistic Regression Analysis | The authors review data on the relationship between TCA plasma concentration and antidepressant effect by using logistic regression analysis. They report that no such association could be found for amitriptyline. For nortriptyline and desipramine, however, a curvilinear relationship was found, while imipramine exhibited a linear association between concentration and effect. |
| DeVane (1987) (30) | Monitoring Cyclic Antidepressants | A curvilinear relationship between concentration and clinical effects in tricyclic antidepressants is supported, with the most reliable evidence for nortriptyline and imipramine. |
| American Psychiatric Association (1984) (31) | Tricyclic Antidepressants – Blood Levels and Clinical Outcomes: An APA Task Force Report | The authors review available evidence on the relationship between concentration and therapeutic effects in tricyclic antidepressant treatment. The results are heterogenous and, except for imipramine, do not support a clear concentration-effect relationship. A linear relationship between the variables was proclaimed for imipramine. |
| Gualtieri et al. (1984) (32) | Blood Level Measurement of Psychoactive Drugs in Pediatric Psychiatry | Evidence on different psychopharmacological substances is reviewed regarding the question whether blood level determinations might be useful in pediatric care. For TCAs, heterogenous findings on concentration-effect relationships are presented. |
| Van Brunt (1983) (33) | The Clinical Utility of Tricyclic Antidepressant Blood Levels: A Review of the Literature | The author summarizes available data on the concentration-effect relationship of different TCAs. While a curvilinear relationship seems to exist for imipramine, nortriptyline, and amitriptyline (+ nortriptyline), evidence is less clear in doxepin, protriptyline, and desipramine. |
| Norman et al. (1982) (34) | Monitoring and Interpretation of Antidepressant Plasma Concentrations | The authors review the relationship between antidepressant (mostly TCA) concentration and clinical response reported in various studies. They conclude that no homogenous statement could be retrieved about the existence and kind of association between the variables based on the literature available at the time. |
| Risch et al. (1981) (35) | Indications and Guideline for Plasma Tricyclic Antidepressant Concentration Monitoring | For TCAs, the authors emphasize the heterogeneity of the available evidence on a potential relationship between concentration and clinical efficacy. Furthermore, they note that there seem to be differences between agents in the nature of the association.  While imipramine and amitriptyline are reported to exhibit a linear relationship, nortriptyline, desipramine, protriptyline, and doxepin show curvilinear or linear relationships in different studies. |
| Levine (1979) (36) | The Role of Plasma Concentrations in the Use of Tricyclic Antidepressant Drugs | The author reviews the results of studies investigating concentration-effect relationships in nortriptyline and amitriptyline treatment. A lack of consensus on the association between the variables is described. Furthermore, methodological properties of studies and differences between studies may prevent unambiguous conclusions. |
| Sjöqvist (1979) (37) | Monitoring of Antidepressant Drug Plasma Levels: The Next Ten Years | Evidence reviewed in this publication points to a curvilinear relationship between nortriptyline concentration and therapeutic effects.  *continued* |
| Orsulak and Schildkraut (1979) (38) | Guidelines for Therapeutic Monitoring of Tricyclic Antidepressant Plasma Levels | The authors review evidence on therapeutic ranges of TCAs and the relationship between concentration and therapeutic response. For imipramine and nortriptyline, therapeutic ranges and a curvilinear relationship between concentration and response are described, while the available data for amitriptyline, protriptyline, doxepin, and desipramine is reported as ambiguous. |
| Kragh-Sörensen et al. (1978) (39) | Relationship Between Antidepressant Effect and Plasma Level of Nortriptyline. Clinical Studies. | Based on three studies investigating the relationship between antidepressant concentration and effect for nortriptyline, the authors propose a therapeutic plasma range between 50 and 150 ng/ml. |
| Gram (1977) (40) | Plasma Level Monitoring of Tricyclic Antidepressant Therapy | Available data on therapeutic ranges and the association between concentration and clinical effect of TCAs is reviewed. Therapeutic ranges seem to be defined for nortriptyline, imipramine, and amitriptyline. However, the author points out that the definitions of upper limits are less homogenous than of lower limits. Additionally, diagnostic differences in participants in primary studies impede homogenous conclusions. Data on protriptyline, maprotiline, and doxepin are described as preliminary and not suitable for review. |

*Note.* Abbreviations TDM therapeutic drug monitoring, TCA tricyclic antidepressants, SSRI selective serotonin reuptake-inhibitors

**Table S2. Search algorithms**

| Randomized controlled trials |
| --- |
| (sulpiride OR quetiapine OR citalopram OR escitalopram OR fluoxetine OR fluvoxamine OR paroxetine OR sertraline OR desvenlafaxine OR duloxetine OR levomilnacipran OR milnacipran OR venlafaxine OR vortioxetine OR trazodone OR reboxetine OR bupropion OR amitriptyline OR amitriptylineoxide OR clomipramine OR desipramine OR dibenzepin OR doxepin OR imipramine OR nortriptyline OR trimipramine OR tianeptine OR maprotiline OR mianserin OR mirtazapine OR tranylcypromine OR moclobemide OR agomelatine OR antidepressant OR dysthymia OR depress*)  AND ("drug level" OR "medication level" OR "drug concentration" OR "blood level" OR "antidepressant concentration" OR "antidepressant level" OR "drug monitoring" OR "serum level" OR "serum concentration" OR "plasma level" OR "plasma concentration")  AND random* |
| Reviews and meta-analyses |
| (sulpiride OR quetiapine OR citalopram OR escitalopram OR fluoxetine OR fluvoxamine OR paroxetine OR sertraline OR desvenlafaxine OR duloxetine OR levomilnacipran OR milnacipran OR venlafaxine OR vortioxetine OR trazodone OR reboxetine OR bupropion OR amitriptyline OR amitriptylineoxide OR clomipramine OR desipramine OR dibenzepin OR doxepin OR imipramine OR nortriptyline OR trimipramine OR tianeptine OR maprotiline OR mianserin OR mirtazapine OR tranylcypromine OR moclobemide OR agomelatine OR antidepressant OR dysthymia OR depress*)  AND ("drug level" OR "medication level" OR "drug concentration" OR "blood level" OR "antidepressant concentration" OR "antidepressant level" OR "drug monitoring" OR "serum level" OR "serum concentration" OR "plasma level" OR "plasma concentration")  AND (review OR "meta-analysis") |

| Table S3. Information on substances and substance classes included in qualitative and quantitative synthesis | | | | | | | |
| --- | --- | --- | --- | --- | --- | --- | --- |
| Substance classes, drugs, and active metabolites ^(1)^ | **Metabolizing enzymes and efflux transporters ^(1)^** | | **Side effects associated with substance class ^(2)^** | **Usual dose in mg/day ^(3)^** | **Elimination half-life in h ^(1)^** | **Therapeutic reference range in ng/ml ^(1)^** | **Level of recommendation for TDM-use ^(1)^** |
| Tricyclic antidepressants | | | | | | | |
| Amitriptyline  (+ NT) | | CYP1A2, CYP2C9, CYP2C19, CYP2D6, CYP3A4, UGT1A3, UGT1A4, UGT2B10,  P-gp (ABCB1) | Cardiovascular:  arrhythmias, orthostatic hypotension, tachycardia  Anticholinergic:  Constipation/obstipation, delirium, dry mouth, urinary hesitancy, visual changes, hypohidrosis, ileus, glaucoma  Neurologic:  myoclonus, seizures  Sexual:  arousal, erectile dysfunction, orgasm dysfunction  Other:  diaphoresis, fall risk, sedation, weight gain, edema, hematopoietic diseases, elevated liver enzymes | 75-300 | 10-28,  18-44 | 80-200 | 1 |
| Amitriptylineoxide (AT  + NT) | | FMO, CYP2C19, CYP2D6 |  | 75-300 | 1.1-2.5,  5-17,  18-44 | 80-200 | 1 |
| Clomipramine  (+ DCL) | | CYP1A2, CYP2C19, CYP2D6, CYP3A4, UGT2B10 |  | 75-250 | 16-60,  37-43 | 230-450 | 1 |
| Desipramine | | CYP2D6 |  | 75-250 | 15-80 | 100-300 | 2 |
| Doxepin  (+ DDOX) | | CYP2C9, CYP2C19, CYP2D6 |  | 75-300 | 15-20 | 50-150 | 2 |
| Imipramine  (+ DMI) | | CYP1A2, CYP2C19, CYP2D6, CYP3A4, UGT1A4, UGT2B10 |  | 75-300 | 11-25,  15-18 | 175-300 | 1 |
| Nortriptyline | | CYP2D6, P-gp (ABCB1) |  | 50-200 | 18-44 | 70-170 | 1 |
| Trimipramine | | CYP2C19, CYP2D6, CYP2C9, CYP3A4, UGT2B10 |  | 75-300 | 23-24 | 150-300 | 2 |
| Selective serotonin reuptake-inhibitors | | | | | | | |
| Citalopram | CYP2C19, CYP2D6, CYP3A4, P-gp (ABCB1) | | Cardiovascular:  arrhythmias, sinus bradycardia  Neurologic:  headaches  Sexual:  arousal, erectile dysfunction, orgasm dysfunction  Other:  activation, akathisia, extrapyramidal side effects, bruxism, diaphoresis, fall risk, gastrointestinal bleeding, insomnia, nausea, vomiting, osteopenia, weight gain, hyponatremia | 20-40 | 38-48 | 50-110 | 1 |
| Escitalopram | CYP2C19, CYP2D6, CYP3A4, P-gp (ABCB1) | |  | 10-20 | 27-32 | 15-80 | 2 |
| Fluoxetine  (+ DFX) | CYP2B6, CYP2C9, CYP2C19, CYP2D6,  P-gp (ABCB1) | |  | 20-40 | 4-6 days,  4-15 days | 120-500 | 3 |
| Fluvoxamine | CYP2D6, CYP1A2,  P-gp (ABCB1) | |  | 100-250 | 21-43 | 60-230 | 2 |
| Paroxetine | CYP2D6, CYP3A4,  P-gp (ABCB1) | |  | 20-40 | 12-44 | 20-65 | 3  *continued* |
| Sertraline | CYP2B6, CYP2C19, CYP2C9, CYP2D6, CYP3A4, UGT1A1,  P-gp (ABCB1) | |  | 50-100 | 22-36 | 10-150 | 2 |
| Tetracyclic antidepressants | | | | | | | |
| Maprotiline | CYP2D6, CYP1A2 | | Cardiovascular:  orthostatic hypotension, tachycardia  Anticholinergic:  dry mouth, urinary hesitancy, visual changes, constipation/obstipation, hypohidrosis, ileus, glaucoma  Other:  weight gain, sedation, nausea, edema, hematopoietic diseases, elevated liver enzymes | 75-225 | 20-58 | 75-130 | 2 |
| Mianserin | CYP2D6, CYP1A2,  CYP3A4 | |  | 60-120 | 14-33 | 15-70 | 2 |
| Selective serotonin-noradrenalin reuptake-inhibitors | | | | | | | |
| Venlafaxine  (+ ODV) | CYP2C19, CYP2D6, CYP2C9, CYP3A4,  P-gp (ABCB1) | | Cardiovascular:  hypertension  Anticholinergic:  dry mouth, constipation/obstipation  Neurologic:  headaches  Sexual:  arousal, erectile dysfunction, orgasm dysfunction  Other:  activation, akathisia, diaphoresis, insomnia, nausea, vomiting, hyponatremia | 75-225 | 4-14,  10-20 | 100-400 | 2 |
| MAO-Inhibitors | | | | | | | |
| Moclobemide | CYP2C19, CYP2D6 | | Cardiovascular:  hypertensive crisis, orthostatic hypotension  Neurologic:  myoclonus, headaches  Sexual:  orgasm dysfunction  Other:  severe serotonin syndrome, weight gain, nausea, gastrointestinal disturbances, insomnia, activation | 300-600 | 2-7 | 300-1000 | 3  *continued* |
| Serotonin-norepinephrine-dopamine reuptake-inhibitors | | | | | | | |
| Bupropion  (+ OH-BUP) | CYP2C19, CYP2B6,  CR | | Cardiovascular:  hypertension  Anticholinergic:  dry mouth, constipation  Neurologic:  headaches, seizures  Other:  insomnia, activation, anxiety, shivering, visual impairments, taste disturbances, tinnitus, fever, epileptic seizures, nausea, vomiting, dermatologic effects, chest pain | 150-300 | 1-15,  17-47 | 850-1500 | 2 |

*Note.* (1) Information retrieved from Hiemke et al. (5) (2) Adverse events reports are combined information retrieved from current guidelines (41, 42) (3) Information on drug dose retrieved from (42) Abbreviations substances: AT Amitriptyline, NT Nortriptyline, DDOX N-desmethyldoxepin, DCL N-desmethyl-clomipramine, DMI Desipramine, DFX N-desmethyl-fluoxetine, ODV O-desmethyl-venlafaxine, OH-BUP Hydroxybupropion, Interpretation of “Level of Recommendation for TDM-use”: 1 strongly recommended, 2 recommended, 3 useful, 4 potentially useful

| Table S4. Qualitative synthesis of 65 studies including 101 treatment arms | | | | | | | | | | | | | |
| --- | --- | --- | --- | --- | --- | --- | --- | --- | --- | --- | --- | --- | --- |
|  |  |  |  |  |  |  | Outcome Assessment | | | Concentration-effect | | Quality Assessment: Numbers of quality criteria rated sufficient | |
| Study | Drug (1) | N  (%♀)  (2) | Mean age | Diagnosis (Classifi-cation system) | Dose:  Mean, range or fixed dose mg/day (SD) | Control  group | Efficacy | Side  effects | | Efficacy | Side  effects | Efficacy  Only  Criteria  1-13 | Efficacy +  Side Effects Criteria  1- 14 |
| Tricyclic Antidepressants | | | | | | |  | | | | | | |
| Amin et al. (1978) (43) | Amitriptyline (+ NT) | 5 | 43,9 | Endogenous  or neurotic depression,  RDC | 100-200 | Active | HAMD |  | | NO |  | 5, 7, 8, 12, 13 |  |
|  | Desipramine | 5 |  |  |  |  |  |  |  |  |  | 5, 7, 8, 12 |  |
| Berm et al. (2016) (44) | Nortriptyline | 40  (73) | 72,2 | MDD,  DSM-IV | 94,5 (30,4) | Active | HAMD-17, MADRS |  | | YES |  | 4, 5, 6, 7, 8, 9, 12, 13 |  |
| Birmaher et al. (1998) (45) | Amitriptyline | 13  (69,2) | 16,2 | MDD,  DSM-III-R | 173,1 (56,3) | Placebo | HDRS, BDI, CGI | CGAS | | NO | NO |  | 1, 4, 5, 6, 7, 8, 9, 14 |
| Breitenstein et al. (2016) (46) | Amitriptyline  Amitriptyline-oxid  Nortriptyline  Trimipramine (combined results) | Tot.  71 | 47,4 | Major recurrent or bipolar depressive episode,  DSM-IV | low dose  150, high  dose 300 | Active;  Dose  group | HAMD-17 | AMDP Somatic Symptoms Scale  (47) | | YES | YES |  | 1, 4, 5, 7, 8, 9, 12, 14 |
| Breyer-Pfaff et al. (1989) (48) | Amitriptyline (+ NT) | 29  (75,9) | 40,9 | Primary depressive  illness, RDC | 150 | Active | HAMD-17 |  | | YES |  | 1, 2, 3, 5, 6, 7, 8, 12, 13 |  |
| Brunswick et al. (1983) (49) | Desipramine | 23  (54) | 43,15 | MDD, RDC | 190 (21) | Active | HDS-17 | ASES | | YES | NO |  | 1, 2, 3, 4, 5, 6, 7, 8, 9, 10, 11, 12, 13, 14 |
|  | Doxepin  (+ DDOX) |  |  |  | 200 |  | HDS-17 | ASES | | NO | NO |  | 1, 2, 3, 4, 5, 6, 7, 8, 9, 10, 11, 12, 14 |
| Burch et al. (1988) (50) | Amitriptyline (+NT) | 68  (70) | 45,2 | Primary depressive  illness, RDC | 40 (3), 109 (10), 202 (10) | Concen-tration  ranges | MADRS |  | | NO |  | 1, 2, 3, 4, 5, 6, 7, 8, 9, 10, 11, 13 | *continued* |
| Burrows et al. (1977) (51) | Nortriptyline | 22  (90) | 41,6 | Primary depressive  illness, RDC | low dose 50, high dose 200 | Dose  group | HDS | Concen-tration level of drop-outs due to side effects | | NO | NO |  | 2, 3, 5, 6, 7, 8, 9 |
| Dahl et al. (1982) (52) | Desipramine | 14  (60) | 43 | MDD, Newcastle-Index II (53) |  | Active | HDS-17 |  | | NO |  | 1, 2, 3, 5, 6, 7, 8, 13 |  |
| De Wilde and Doogan (1982) (54) | Clomipramine (+ DCL) | 14 (33,3) | 45,8 | Depressive  illness, RDC | 241,1 | Active | HRSD-17 & CGI | EKG, BP,  pulse, hae-matology,  blood bio-chemistry,  reported  unwanted  effects | | NO | NO |  | 3, 4, 5, 6, 7, 8, 9, 12, 13, 14 |
| De Wilde et al. (1983) (55) | Clomipramine (+ DCL) | 20 | ? | Depressive  illness, RDC | 109-144 | Active | HRSD-17 & CGI |  | | NO |  | 4, 5, 6, 8, 9, 12 |  |
| DUAG (1986) (56) | Clomipramine (+ DCL) | 52  (69,6) | ? | Endogenous & Non-endogenous depression,  Newcastle Inventory (57) | 150 | Active | HDS-17 |  | | NO |  | 1, 2, 3, 5, 7, 8, 9, 10, 11, 12 |  |
| DUAG (1990) (58) | Clomipramine (+ DCL) | 41  (67,6) | ? | MDD,  DSM-III | 150 | Active | HDS-17 | UKU (59) | | NO | NO |  | 1, 2, 3, 4, 5, 7, 8, 9, 10, 11, 12, 13, 14 |
| DUAG (1993) (60) | Clomipramine (+ DCL) | 50  (58,6) | ? | MDD,  ICD-10 | 75 | Active | HAMD-17 |  | | NO |  | 1, 2, 3, 4, 5, 6, 7, 8, 9, 10, 11,  12, 13 |  |
| DUAG (1999) (61) | Clomipramine (+DCL) | 126  (66) | ? | Primary depression,  DSM-III-R | 25, 50, 75, 125, 200 | Dose  group | HDS-17 | BP, UKU | | YES | YES |  | 1, 2, 3, 4, 5, 6, 7, 8, 9, 10, 12, 13, 14 |
| Feet et al (1987) (62) | Imipramine  (+ DMI) | 52 | ? | Non-agitated depression,  RDC | 100-200 | Aug-men-tation: Active vs. Placebo | MADRS | VAS Global Side Effects (DMI) | | NO | YES |  | 1, 3, 4, 5, 7, 8, 13  *continued* |
| Geller et al. (1990) (63) | Nortriptyline | 12  (25) | 14 | MDD, RDC,  DSM-III | 85 | Placebo | CDRS |  | | Inverse |  | 1, 2, 3, 4, 5, 6, 7, 8, 9, 10, 11, 12, 13 |  |
| Geller et al. (1992) (64) | Nortriptyline | 26  (26,9) | 9,7 | MDD, RDC,  DSM-III | 10-140 | Placebo | CDRS |  | | NO |  | 1, 2, 3, 4, 5, 6, 7, 8, 9, 10, 11, 12, 13 |  |
| Gwirtsman et al. (1983) (65) | Doxepin | 18  (8,2) | 63,8 | MD,  DSM-III | 129 (9,32) | Active | HAMD-21 |  | | NO |  | 1, 3, 4, 5, 6, 7, 8, 13 |  |
| Hodgson et al. (2014) (66) | Nortriptyline | 191 (62,9) | 41,98 (7) | Unipolar depression,  ICD-10/  DSM-IV | 104,94 (31,35) | Active | MADRS |  | | NO |  | 1, 2, 3, 4, 5, 6, 8, 9, 13 |  |
| Hrdina et al. (1988) (67) | Doxepin  (+ DDOX) | 13 (73,3) | 41,2 | Major  depression,  DSM-III | 75-225 | Active | HAMD-21 | Side effects checklist | | Inverse | YES |  | 1, 2, 3, 4, 5, 7, 8, 9, 10, 12 |
| Klok et al. (1981) (68) | Clomipramine (+ DCL) | 13  (100) | 46,5 | Endogenous  depression | 150 | Active | HRSD-17 | 23-item checklist | | NO | NO |  | 1, 3, 5, 6, 7, 8, 9, 12, 13, 14 |
| Kragh-Sorensen et al. (1976) (69) | Nortriptyline | 23  (78,3) | 45,43 | Endogenous depression,  (57) | 156,92 | Con-cen-  tration  ranges | CDRS | Side effects  checklist,  ECG | | Inverse | NO |  | 1, 2, 3, 4, 5, 6, 7, 8, 9, 10, 11, 14 |
| Kutcher et al. (1994) (70) | Desipramine | 42  (64) | 17,6 | MDD,  DSM-III-R | 200 | Placebo | HAMD |  | | NO |  | 3, 4, 5, 6, 7, 8, 9, 10, 11, 12, 13 |  |
| Lehmann et al. (1982) (71) | Amitriptyline (+ NT) | 8  (13) | 44,1 | Unipolar  MDD, RDC | 131 | Active | HRS |  | | NO |  | 1, 2, 3, 4, 5, 6, 7, 9, 10, 11, 12, 13 |  |
|  | Nortriptyline | 10  (50) |  |  | 95 |  | HRS |  |  | YES |  |  |  |
| Linnoila et al. (1980) (72) | Clomipramine (+ DCL) | 13  (71) | 39,4 | Primary depressive disorder | 75 | Active | HAMD-21 |  | | YES |  | 1, 3, 5, 7, 8, 9, 12, 13 |  |
|  | Doxepin  (+ DDOX) | 13  (71) |  |  | 75 |  |  |  |  | NO |  |  |  |
| Matuzas et al. (1982) (73) | Imipramine  (+ DMI) | 10 | ? | Depressive illness,  RDC | 150 | Placebo | HAMD, GSS, GCS, BADS, HSCL |  | | YES |  | 1, 3, 4, 5, 8, 12 |  |
| McCue et al. (1989) (74) | Nortriptyline | 64  (64) | 64,3 | Unipolar  MDD, RDC | 71,2 (20,7) | Placebo | HAMD-21 | TESS, BP | | NO | NO |  | 1, 2, 3, 4, 5, 7, 8, 13, 14 |
| Mendlewicz et al. (1980) (75) | Amitriptyline (+ NT) | 16  (75) | 40,7 | Primary depressive illness, RDC | 200 | Active | HAMD | 21-item  side effects questionn-aire | | NO | NO |  | 1, 3, 5, 6, 7, 8, 10, 11, 12, 13, 14  *continued* |
| Monteleone and Fabrazzo (1994) (76) | Amitriptyline (+ NT) | 10  (0) | 66.1 | Major depression, DSM-III-R | 75 | Active | HAMD-17 | TESS | | NO | NO |  | 1, 2, 3, 4, 5, 6, 7, 8, 9, 10, 11, 12, 13, 14 |
| Montgomery et al. (1983) (77) | Imipramine  (+ DMI) | 10 (77) | 50 | Primary affective disorder,  RDC | 150 | Active | HAMD-17, MADRS |  | | NO |  | 1, 2, 3, 4, 5, 6, 8, 10, 11, 12, 13 |  |
| Montgomery et al. (1980) (78) | Amitriptyline (+ NT) | 17 (72) | 42,8 | Primary depressive illness, RDC | 150 | Active | HAMD | Side effects  checklist | | NO | NO |  | 1, 2, 3, 4, 5, 6, 7, 8, 10, 11, 12, 13, 14 |
| Moyes et al. (1980) (79) | Amitriptyline (+ NT) | 48 (73) | ? | Depression | 75-150 | Active | HRS, BDI |  | | YES |  | 1, 2, 3, 5, 7, 8, 9, 10, 12 |  |
|  | Clomipramine (+ DCL) |  |  |  |  |  | HAMD, BDI | Side effects scores | | NO | NO |  | 1, 2, 3, 5, 7, 8, 9, 10, 12, 14 |
| Mulgirigama et al. (1977) (80) | Clomipramine | 41 (58) | 41 | Endogenous or neurotic/  reactive depression | 150 | Active | Depression observer-rating scale (Mulgirigama, 1977) (80) |  | | NO |  | 1, 8, 9, 12 |  |
| Müller et al. (2003) (81) | Amitriptyline (+ NT) | 69 (63) | 49,9 | Major depression, Bipolar depression, Dysthymia,  Depressive  adjustment disorder,  DSM-IV | 126 (35) | TDM  vs.  No  TDM | HAMD-17,  CGI | UKU | | YES | YES |  | 1, 2, 3, 4, 5, 6, 8, 13, 14 |
|  | Doxepin  (+ DDOX) | 15  (63) |  |  | 155 (47) |  | HAMD-17, CGI |  | | YES |  |  |  |
| Müller-Oerlinghausen and Fähndrich (1985) (82) | Clomipramine (+ DCL) | 30  (80) | 50 | ? | 100 | Active | HDRS |  | | NO |  | 3, 5, 7, 8, 12, 13 |  |
| Nathan et al. (1990) (83) | Desipramine | 17  (78) | 39,6 | Major depression, DSM-III | 206 (62) | Active | HAMD-17 |  | | YES |  |  | 1, 4, 5, 6, 7, 8, 10, 12, 13, 14 |
| Pollock et al. (1993) (84) | Clomipramine (+ DCL) | 32  (63) | 39,6 | MDD, RDC | 50-200 | Oral vs.  IV | HAMD-17 | RSSE | | NO | NO |  | 1, 3, 4, 5, 6, 8, 10, 11, 13, 14 |
| Robinson et al. (1985) (85) | Amitriptyline (+NT) | 80 | ? | MDD or  atypical depression, DSM-III | 150 | Active | HRS-17 |  | | NO |  | 1, 2, 4, 5, 6, 8, 12, 13 | *continued* |
| Simpson et al. (1982) (86) | Imipramine (+ DMI) | 27  (67) | 42,3 | MDD, RDC | 100 | Active | Zung Self Rating Depression Scale (87), BDRS, CGI |  | | YES |  | 1, 3, 4, 8, 12 |  |
| Streim et al. (2000) (88) | Nortriptyline | 41  (33) | 79,49 | MDD, Dysthymia, Minor depression, DSM-IV | 13, high dose 80 | Dose  groups | HAMD-21 |  | | YES |  | 1, 2, 3, 4, 5, 6, 7, 8, 9, 10, 11, 13 |  |
| Thomson et al. (1982) (89) | Amitriptyline (+NT) | 37  (22) | ? | Major affective disorder, RDC | 150 | Active, Placebo | HDS-18 |  | | NO |  | 1, 3, 4, 5, 6, 7, 8, 9, 10, 12, 13 |  |
| Vandel et al. (1982) (90) | Clomipramine (+ DCL) | 28  (58) | 44 | Endogenous or non-endogenous depressive syndrome | 75-225 | Dose  groups | HRS |  | | Inverse |  | 1, 2, 3, 5, 8, 9, 13 |  |
| Veith et al. (1983) (91) | Desipramine | 26  (54) | 35 | Primary unipolar affective disorder,  RDC | 100-200 | Active | HDS |  | | YES |  | 1, 3, 4, 5, 6, 7, 8, 9, 13 |  |
|  | Amitriptyline (+ NT) | 20  (30) |  |  |  |  |  |  |  | NO |  |  |  |
| Veith et al. (1980) (92) (5) | Desipramine | 26  (54) | 36 | Primary unipolar affective disorder, RDC | 100-200 | Active |  | Adverse effects, ECG, resting pulse, supine BP | | --- | YES |  | 1, 3, 4, 8, 9, 12, 13, 14 |
| Ziegler et al. (1977) (93) | Amitriptyline (+ NT) | 16  (63) | 32,2 | Primary or secondary affective disorder,  RDC | 119 (3) | Active | HRS-17 |  | | YES |  | 1, 2, 3, 4, 5, 7, 8, 12, 13 |  |
|  | Nortriptyline | 9  (67) |  |  | 117 (3) |  | HRS-17 |  | | Inverse |  |  |  |
| Selective serotonin reuptake-inhibitors | | | | | | |  | | | | | | |
| Allard et al. (2004) (94) | Citalopram | 42  (80) | 73 | MD, DSM-IV | 10-20 | Active | MADRS |  | | NO |  | 4, 5, 6, 7, 8, 9, 12, 13 |  |
| Bjerkenstedt et al. (1985) (95) | Citalopram | 23  (73) | ? | Depressive illness, Newcastle Inventory | 5,25,50 | Dose  groups | MADRS |  | | NO |  | 1, 2, 3, 5, 7, 8, 9, 12, 13 | *continued* |
| Breitenstein et al. (2016) (46) | Citalopram | Tot.  71 | 47,4 | Major recurrent or bipolar depressive episode,  DSM-IV | low dose 40,  normal dose 80 | Active;  Dose  group | HAMD-17 | AMDP Somatic Symptoms Scale | | YES | YES |  | 1, 4, 5, 7, 8, 9, 12, 14 |
|  | Escitalopram |  |  |  | 20 vs. 40 |  |  |  |  |  |  |  |  |
|  | Paroxetine |  |  |  | 40 vs. 70 |  |  |  |  |  |  |  |  |
|  | Sertraline |  |  |  | 100 vs. 200 |  |  |  |  |  |  |  |  |
| De Wilde and Doogan (1982) (54) | Fluvoxamine | 11  (73) | 45,8 | Depressive illness,  RDC | 265,9 | Active | HRSD-17 & CGI | EKG, BP,  pulse,  haematolo-gy, blood biochemis-try unwan-ted effects reported in interview | | Inverse | NO |  | 3, 4, 5, 6, 7, 8, 9, 12, 14 |
| De Wilde et al. (1983) (55) | Fluvoxamine | 20 | ? | Depressive illness,RDC | 223-300 | Active | HRSD-17, CGI |  | | NO |  | 4, 5, 6, 8, 9, 12 |  |
| De Wilde et al. (1985) (96) | Citalopram | 14  (53) | 46,8 | Endogenous Depression, RDC | 40-80 | Active | CGI | Side effects checklist | | NO | NO |  | 2, 3, 4, 5, 7, 8, 9, 14 |
| DUAG (1990) (58) | Paroxetine | 53  (68) | ? | MDD, DSM-III | 30 | Active | HDS-17 | UKU | | NO | NO |  | 1, 2, 3, 4, 5, 7,  8, 9, 10, 12,  13, 14 |
| DUAG (1986) (56) | Citalopram | 50  (70) | ? | Endogenous & Non-endogenous depression,  Newcastle Inventory | 40 | Active | HDS-17 |  | | NO |  | 1, 2, 3, 5, 7, 8, 9, 10, 11, 12 |  |
| Ghose (1997) (97) | Paroxetine | 12  (66, 33) | 78,7 | Reactive or  endogenous  unipolar depression | 15 vs. 30 | Dose  group | HRS-17 | Side effects checklist (98) | | NO | NO |  | 1, 3, 5, 6, 8,  9, 10, 11,  12, 14 |
| Hebenstreit et al. (1989) (99) | Paroxetine | 44 | ? | Major depression, DSM-III | 20-50 | Dose  group | HAMD | 24-item symptom checklist | | NO | NO |  | 1, 2, 3, 4, 5,  8, 9, 12,  13, 14 |
| Hodgson et al. (2014) (66) | Escitalopram | 266  (63) | 41,9 | Unipolar depression, ICD-10/DSM-IV | 16,44 (6,44) | Active | MADRS |  | | Inverse |  | 1, 2, 3, 4, 5, 6, 8, 9, 13 | *continued* |
| Kasper et al. (1993) (100) | Fluvoxamine | 18  (75) | 47,5 | MDD, RDC &  DSM-III | 229 (47) | Active | HDRS-21 | AMDP system for somatic symptoms | | NO | YES |  | 1, 3, 4, 5, 6,  7, 8, 9, 10,  11, 13, 14 |
| Kelly et al. (1989) (101) | Fluoxetine  (+ DFX) | 13  (70) | 40 | MDD, DSM-III | 51 (16) | Active | HRSD, CGI |  | | NO |  | 1, 3, 4, 5, 6, 7, 8, 9, 10, 11 |  |
| Klok et al. (1981) (68) | Fluvoxamine | 10  (10) | 46,5 | Endogenous  depression | 150 | Active | HRSD-17 | 23-item checklist of  unwanted effects | | NO | NO |  | 1, 3, 5, 6,  7, 8, 9, 12,  13, 14 |
| Leuchter et al. (2009) (102) | Escitalopram | 73  (67) | 42,9 | MDD, DSM-IV | 10 | Active | HAMD-17 |  | | NO |  | 1, 4, 5, 6, 7, 8, 9, 12 |  |
| Nathan et al. (1990) (83) | Fluvoxamine | 16  (77) | 39,8 | Major depression, DSM-III | 203 (70) | Active | HAMD-17 |  | | YES |  | 1, 4, 5, 6, 7, 8, 10, 12, 13 |  |
| Perez et al. (2001) (103) | Fluoxetine  (+ DFX) | 111 (62,80) | 43,4 | MDD, DSM-IV | 20 | Aug-men-tation:  Active vs. Placebo | HAMD-17 |  | | NO |  | 1, 2, 3, 4, 5, 6, 7, 8, 10, 11, 12, 13 |  |
| Preskorn et al. (1991) (104) | Fluoxetine (+DFX) | 24 | ? | MD, DSM-III | 20-60 | Active | HAMD |  | | NO |  | 1, 4, 5, 6, 8, 9, 10, 11, 13 |  |
| Reis et al. (2004) (105) | Sertraline  (+ DSERT) | 70  (63) | ? | MDD, DSM-III-R | 50-150 | Active | MADRS | |  | NO |  |  | 1, 3, 4, 5, 6, 7, 8, 9, 12, 13, 14 |
|  | Paroxetine | 70  (73) |  |  | 20-40 |  |  |  |  |  |  |  | 1, 2, 3, 4, 5, 6, 7, 8, 9, 12, 13, 14 |
| Sakolsky et al. (2011) (106) | Citalopram | 27 | ? | MDD, DSM-IV | 31,2 (10,1) | Active | CGI, CDRS | | SEFCA (107) | NO | NO |  | 1, 3, 4, 5, 7, 8, 9, 13, 14 |
|  | Fluoxetine  (+ DFX) | 64 |  |  | 33,8 (9,3) |  |  |  |  |  |  |  |  |
|  | Paroxetine | 34 |  |  | 35,2 (8,7) |  |  |  |  |  |  |  |  |
| Simeon et al. (1990) (108) | Fluoxetine (+DFX) | 40 | ? | MD, DSM-III | ? | Placebo | HAMD, CGI |  | | NO |  | 4, 5, 8, 10, 11 |  |
| Tetracyclic Antidepressants | | | | | | |  | | | | | | |
| Gwirtsman et al. (1983) (65) | Maprotiline | 20 (8) | 63,8 | Major depression, DSM-III | 144 (9,31) | Active | HAMD-21 |  | | NO |  | 1, 3, 4, 5, 6, 7, 8, 13 | *continued* |
| Hrdina and Lapierre (1986) (109) | Maprotiline | 26 | ? | MDD, DSM-III-R | 180 | Active | HAMD-22 |  | | NO |  | 1, 3, 4, 5, 7, 8, 9, 10 |  |
| Kasper et al. (1993) (100) | Maprotiline | 14  (75) | 47,5 | MDD, DSM-III | 236 (32) | Active | HDRS-21 | AMDP system for somatic symptoms | | NO | YES |  | 1, 3, 4, 5, 6,  7, 8, 9, 10,  11, 13, 14 |
| Miller et al. (1977) (110) | Maprotiline | 12  (67) | 41,4 | Depressive illness | 75 | Dose  groups | 17 Item depression rating scale | Voluntary report | | NO | NO |  | 3, 6, 8, 9, 12, 13 |
| Monteleone and Fabrazzo (1994) (76) | Mianserin | 10  (0) | 66,4 | Major depression, DSM-III-R | 60 | Active | HAMD-17 | TESS | | YES | NO |  | 1, 2, 3, 4, 5, 6, 7, 8, 9, 10, 11, 12, 13, 14 |
| Montgomery et al. (1983) (77) | Mianserin | 10  (92) | 46 | Primary affective disorder, RDC | 60 | Active | HAMD-17, MADRS |  | | NO |  | 1, 2, 3, 4, 5, 6, 8, 10, 11, 12, 13 |  |
| Montgomery et al. (1978) (111) | Mianserin | 14  (74) | 44,4 | Primary depressive illness,RDC | 60 | Dose  groups | MADRS, HRS-17 | Reports of side effects | | Inverse | NO |  | 1, 2, 3, 4, 5, 6, 7, 8, 9, 11, 12, 14 |
| Montgomery et al. (1980) (78) | Maprotiline | 17  (67) | 42,8 | Primary depressive illness, RDC | 150 | Active | MADRS, HAMD, BSRI | Side effects checklist | | NO | NO |  | 1, 2, 3, 4, 5, 6, 7, 8, 10, 11, 12, 14 |
| Müller-Örlinghausen and Fähndrich (1985) (82) | Maprotiline | 30  (70) | 47,5 | ? | 100 | Active | HDRS |  | | NO |  | 1, 3, 5, 7, 8, 12, 13 |  |
| Selective serotonin-noradrenaline-reuptake inhibitors (Venlafaxine) | | | | | | |  | | | | | | |
| Allard et al. (2004) (94) | Venlafaxine (+ ODV) | 45  (80) | 73 | MD, DSM-IV | 75-150 | Active | MADRS |  | | NO |  | 4, 5, 6, 7, 8, 9, 12, 13 |  |
| Berm et al. (2016) (44) | Venlafaxine (+ ODV) | 35  (73) | 72,2 | MDD, DSM-IV | 156 (71) | Active | HAMD-17, MADRS |  | | NO |  | 4, 5, 6, 7, 8, 9, 12, 13 |  |
| Breitenstein et al. (2016) (46) | Venlafaxine | 71 | 47,4 | Major recurrent or bipolar depressive episode, DSM-IV | low dose 225, standard dose 450 | Active;  Dose  groups | HAMD-17 | AMDP Somatic Symptoms Scale | | YES | YES |  | 1, 4, 5, 7, 8, 9, 12, 14 |
| Lobello et al. (2010) (112) (3) | Venlafaxine (+ ODV) | 464  (61) | 41,2 | MD,  DSM-III/-R,  MDD, DSM-IV | EM: 129,08 (68,30), PM: 128,6 (60,47) | Placebo | HDRS-17, HDRS-6, MADRS,  CGI-S,  CGI-I |  | | NO |  | 3, 4, 5, 6, 7, 8, 9, 10, 11 | *continued* |
| Martiny et al. (2012) (113) | Venlafaxine (+ ODV) | 26  (60, 31) | 46,9 | MDD, DSM-IV | 150 | Augmen-  tation: Active vs. Placebo | HAMD-17 |  | | NO |  | 1, 3, 4, 5, 6, 7, 8, 12, 13 |  |
| Sakolsky et al. (2011) (106) | Venlafaxine  (+ ODV) | 119 | ? | MDD, DSM-IV | 200,9 (35,2) | Active | CGI, CDRS | SEFCA, dizziness, cardiovas-cular & dermato-logical AE | | NO | YES |  | 1, 3, 4, 5, 7, 8, 9, 13, 14 |
| MAO-Inhibitors | | | | | | |  | | | | | | |
| DUAG (1993) (60) | Moclobemide | 42  (70) | ? | MDD, ICD-10 | 400 | Active | HAMD-17 |  | | NO |  | 1, 3, 4, 5, 6, 7, 8, 9, 10, 11, 12, 13 |  |
| Serotonin-norepinephrine-dopamine reuptake inhibitors (Bupropion) | | | | | | |  | | | | | | |
| Fogel et al. (1984) (114) (4) | Bupropion | 15 | ? | Psychiatrist rated depression | ? | Active | HAMD, Hamilton Anxiety test, CGI, BDI, Zung Self-Rating |  | | NO |  | 1, 3, 5, 7, 8, 10, 11, 12, 13 |  |

*Note.* Table design based on Kloosterboer et al. (2). YES descriptions indicate an association in the expected direction: higher concentration values associated with greater response/more or more severe side effects, Inverse descriptions indicate the reverse association: higher concentrations associated with worse outcomes, NO indicates the absence of an association. Abbreviations substances: NT Nortriptyline, DDOX N-desmethyldoxepin, DCL N-desmethyl-clomipramine, DMI Desipramine, DFX N-desmethyl-fluoxetine, ODV O-desmethyl-venlafaxine Abbreviations Diagnoses/Classifications: RDC Research Diagnostic Criteria (115), DSM Diagnostic and Statistical Manual of Mental Disorders (American Psychiaric Association [APA]), ICD International Classification of Diseases (World Health Organization [WHO]), MDD Major Depressive Disorder, MD Major Depression. Abbreviations Outcome Assessment: AMDP Arbeitsgemeinschaft für Methoden und Dokumentation in der Psychiatrie, ASES Asberg Side Effects (116), TESS Treatment Emergent Side Effects Scale (117), (HAMD/HRS/HRSD/HDRS Hamilton Rating Scale for Depression (118), CGI Clinical Global Impression Scale (119), MADRS Montgomery Asberg Depression Rating Scale (120), BDRS Brief Depression Rating Scale (121), BDI/BSRI Beck Depression Inventory/Beck Self Ratings Inventory (122), TRR Therapeutic Reference Range, CGAS Children’s Global Assessment Scale (123), CDRS Children’s Depression rating Scale (124), ECG Electrocardiogram, BP Blood Pressure, VAS Visual analogue scale, RSSE Rating Scale of Side Effects (World Health Organization [WHO], 1986), GSS Global Status Scale (73), GCS Global Change Scale (73), BADS Brief Anxiety/Depression Scale (73), HSCL Hopkins Symptom Checklist (125), SEFCA Side Effects Form for Children and Adolescents (107), GM Geometric mean, DUAG: Danish University Antidepressant group, Hamilton Rating Scale: Number of items differs, specified as e.g. HAMD-XX, whenever reported by authors (1) Whenever concentration-effect relationships were reported for the sum of drugs and their metabolites, the metabolites are given in parentheses. (2) % female is given for the total sample, unless there were specific information for the treatment groups given in the study. Numbers are rounded.

(3) Lobello et al^.^ (112) was a meta-analysis of four randomized-controlled trials investigating a concentration-effect relationship, Lobello quality assessment: all 4 primary studies checked for criteria, if one of the studies did not fulfill the requirements, the entire criterion was considered insufficient. (4) Fogel et al. (114) report mean concentrations of 40 ng/ml, which is approximately 20 times smaller than the therapeutic reference range for Bupropion (5). (5) Veith et al. (91, 92) possibly included data from the same sample treated with desipramine, but reported different outcomes.

| Table S5. Quantitative synthesis of 27 treatment arms from 19 studies | | | | | | | | |
| --- | --- | --- | --- | --- | --- | --- | --- | --- |
| Means & standard deviations of antidepressant concentration in ng/ml | | | | | | | | |
|  |  | **Responders** | | | **Non-responders** | | |  |
| Study | Substance  (+ active metabolite) | Mean | SD | N | Mean | SD | N | Information on concentration data & response criteria |
| Breyer-Pfaff et al. (1989) (48) | Amitriptyline (+ NT) | 149,6 | 50 | 19 | 163,4 | 67 | 10 | Calculated manually: day 28, HAMD 50% |
| Brunswick et al. (1983) (49) | Desipramine | 237,1 | 165,9 | 15 | 225,6 | 192,2 | 8 | Calculated manually, week 4, response criterion assigned to HAMD 50% |
|  | Doxepin  (+ DDOX) | 191,7 | 196,6 | 11 | 147,5 | 41,4 | 4 |  |
| Burrows et al. (1977) (51) | Nortriptyline | 108,3 | 75,6 | 7 | 197,1 | 115,3 | 15 | Calculated manually, week 4, HAMD 50% |
| De Wilde and Doogan (1982) (54) | Clomipramine (+ DCL) | 566 | 618.2 | 9 | 356 | 346,3 | 2 | Calculated manually, week 4, HAMD 50% |
|  | Fluvoxamine | 427,6 | 259,3 | 9 | 906 | 12 | 5 |  |
| Fogel et al. (1984) (114) | Bupropion | 44,8 | 14,3 | 9 | 24,7 | 7,6 | 4 | Values given in study, response criterion: sum of change on all tests included in the study > 60 |
| Geller et al. (1992) (64) | Nortriptyline | 87,3 | 11,8 | 8 | 91,1 | 15,5 | 18 | Values given in study, week 8, CDRS $\leq$ 20 |
| Hrdina et al. (1988) (67) | Doxepin  (+ DDOX) | 114,0 | 24,0 | 7 | 94,0 | 8,0 | 6 | Values given in study, day 28, HAMD 50% |
| Kelly et al. (1989) (101) | Fluoxetine  (+ DFX) | 413,9 | 185,5 | 10 | 624,3 | 100,6 | 3 | Calculated manually, week 6, CGI 1 or 2 in week 6 |
| Kragh-Sorensen et al. (1976) (69) | Nortriptyline | 122,2 | 33,8 | 17 | 217,0 | 25,4 | 6 | Calculated manually, week 6, response criterion final Cronholm-Ottoson-Rating Scale (126) value $\leq$ 3 |
| Lehmann et al. (1982) (71) | Amitriptyline (+ NT) | 188,0 | 37,5 | 5 | 110,0 | 38,6 | 3 | Calculated manually: week 3, HAMD $\leq$ 12 |
|  | Nortriptyline | 156,0 | 43,2 | 7 | 147,0 | 4,3 | 3 |  |
| Leuchter et al. (2009) (102) | Escitalopram | 17,6 | 7,8 | 38 | 19,5 | 8,6 | 35 | Values given in study,  Day 49, HAMD 50% |
| Linnoila et al. (1980) (72) | Clomipramine (+ DCL) | 102,9 | 42,3 | 5 | 62,3 | 42,4 | 8 | Values given in study,  week 3, HAMD < 10 |
|  | Doxepin  (+ DDOX) | 18,5 | 11,2 | 7 | 8,1 | 9,7 | 6 |  |
| Martiny et al. (2012) (113) | Venlafaxine  (+ ODV) | 420,8 | 151,3 | 22 | 297,9 | 95,3 | 6 | Values given in study,  day 19, HAMD 50% (1) |
| McCue et al. (1989) (74) | Nortriptyline | 94,3 | 36,7 | 33 | 102,6 | 49,5 | 31 | Values given in study,  week 7, HAMD < 10 |
| Mendlewicz et al. (1980) (75) | Amitriptyline (+ NT) | 186,6 | 82,7 | 11 | 187,8 | 42,9 | 7 | Calculated manually, day 24, response criterion assigned to HAMD 50% |
| Müller-Örlinghausen and Fähndrich (1985) (82) | Maprotiline | 77,0 | 56,0 | 21 | 84,0 | 50,0 | 9 | Values given in study,  week 3, HAMD 50%  *continued* |
| Nathan et al. (1990) (83) | Desipramine | 173,11 | 53,42 | 9 | 183,13 | 40,92 | 8 | Values given in study,  day 27, HAMD < 10 |
|  | Fluvoxamine | 311,64 | 148,82 | 11 | 213,0 | 73,31 | 5 |  |
| Sakolsky et al. (2011) (106) | Citalopram | 57,6 | 37,6 | 16 | 40,2 | 35,2 | 11 | Values given in study, week 12, CGI-I $\leq$2, CDRS-R decrease $\geq$ 50%, CDRS-R $\leq$40 |
|  | Fluoxetine | 193,9 | 68,8 | 32 | 170,4 | 108,2 | 32 |  |
|  | Paroxetine | 29,1 | 28,4 | 13 | 37,1 | 29,7 | 21 |  |
|  | Venlafaxine  (+ ODV) | 293,0 | 175,6 | 65 | 360,2 | 212,2 | 54 |  |
| Vandel et al. (1982) (90) | Clomipramine (+ DCL) | 218,3 | 169,0 | 18 | 367,4 | 202,0 | 10 | Values given in study, Day 28 HAMD 50% |

*Note.* Abbreviations: NT Nortriptyline, DDOX N-desmethyldoxepin, DCL N-desmethyl-clomipramine, DFX N-desmethyl-fluoxetine, ODV O-desmethyl-venlafaxine, HAMD Hamilton Rating Scale for Depression (118), CDRS Children’s Depression rating Scale (124), CGI Clinical Global Impression Scale (119).

(1) Concentration values originally reported as nmol/l, manually converted to ng/ml as described in Hiemke et al. (5).

**Table S6. Sensitivity analyses**

| Sensitivity analyses (N treatment arms included) | SMD | CI (95%) | P | I^2^ |
| --- | --- | --- | --- | --- |
| Without any selection, reference effect (27) | -0,05 | [-0,31, 0,21] | 0,70 | 59% |
| Elimination of study with highest weight (26) | -0,03 | [-0,01, 0,25] | 0,83 | 59% |
| Unipolar patients only (23) | -0,01 | [-0,28, 0,27] | 0,97 | 60% |
| HAMD 50% reduction as response criterion (10) | -0,17 | [-0,53, 0,19] | 0,34 | 36% |
| HAMD scale for response-assessment (16) | 0,02 | [-0,27, 0,31] | 0,88 | 45% |
| RoB 2.0 high risk studies excluded (4) | -0,08 | [-1,43, 1,27] | 0,91 | 77% |
| Concentration-efficacy relationship primary vs. secondary endpoint (secondary endpoint excluded) (15) | -0,09 | [-0,44, 0,26] | 0,61 | 63% |
| Mean Concentrations (both responder and non-responders) within TRR (14) | 0,12 | [-0,29, 0,06] | 0,20 | 0% |

*Note.* Abbreviations: CI confidence interval, HAMD Hamilton Depression Rating Scale (118), TRR therapeutic reference range (5), RoB 2.0 Cochrane Risk of bias tool 2.0 (127).

| Table S7. Percent of quality assessment criteria rated sufficient in studies sorted by decade of publication | | | | |
| --- | --- | --- | --- | --- |
| Study | **Substance** | | **Quality Assessment: Numbers of quality criteria rated sufficient** | **% Quality assessment criteria rated sufficient** |
| 1970s | | | | |
| Amin et al. (1978) (43) | | Amitriptyline (+ NT) | 5, 7, 8, 12, 13 | 38.5 |
|  |  | Desipramine | 5, 7, 8, 12 | 30.8 |
| Burrows et al. (1977) (51) | | Nortriptyline | 2, 3, 5, 6, 7, 8, 9 | 50 |
| Kragh-Sörensen et al. (1976) (69) | | Nortriptyline | 1, 2, 3, 4, 5, 6, 7, 8, 9, 10, 11, 14 | 85.7 |
| Miller et al. (1977) (110) | | Maprotiline | 3, 6, 8, 9, 12, 13 | 42.9 |
| Montgomery et al. (1978) (111) | | Mianserin | 1, 2, 3, 4, 5, 6, 7, 8, 9, 11, 12, 14 | 85.7 |
| Mulgirigama et al. (1977) (80) | | Clomipramine | 1, 8, 9, 12 | 30.8 |
| Ziegler et al. (1977) (93) | | Amitriptyline (+ NT) | 1, 2, 3, 4, 5, 7, 8, 12, 13 | 69.2 |
|  |  | Nortriptyline |  |  |
| Mean 1970s: 50.9 | | | | |
| 1980s | | | | |
| Bjerkenstedt et al. (1985) (95) | | Citalopram | 1, 2, 3, 5, 7, 8, 9, 12, 13 | 69.2 |
| Breyer-Pfaff et al. (1989) (48) | | Amitriptyline (+ NT) | 1, 2, 3, 5, 6, 7, 8, 12, 13 | 69.2 |
| Brunswick et al. (1983) (49) | | Desipramine | 1, 2, 3, 4, 5, 6, 7, 8, 9, 10, 11, 12, 13, 14 | 100 |
|  |  | Doxepin (+DDOX) | 1, 2, 3, 4, 5, 6, 7, 8, 9, 10, 11, 12, 14 | 92.9 |
| Burch et al. (1988) (50) | | Amitriptyline (+ NT) | 1, 2, 3, 4, 5, 6, 7, 8, 9, 10, 11, 13 | 92.3 |
| Dahl et al. (1982) (52) | | Desipramine | 1, 2, 3, 5, 6, 7, 8, 13 | 61.5 |
| De Wilde and Doogan (1982) (54) | | Clomipramine (+ DCL) | 3, 4, 5, 6, 7, 8, 9, 12, 13, 14 | 71.4 |
|  |  | Fluvoxamine | 3, 4, 5, 6, 7, 8, 9, 12, 14 | 64.3 |
| De Wilde et al. (1983) (55) | | Clomipramine (+ DCL) | 4, 5, 6, 8, 9, 12 | 46.2 |
|  |  | Fluvoxamine |  |  |
| De Wilde et al. (1985) (96) | | Citalopram | 2, 3, 4, 5, 7, 8, 9, 14 | 57.1 |
| DUAG (1986) (56) | | Clomipramine (+ DCL) | 1, 2, 3, 5, 7, 8, 9, 10, 11, 12 | 76.9 |
|  |  | Citalopram |  |  |
| Feet et al. (1987) (62) | | Imipramine (+ DMI) | 1, 3, 4, 5, 7, 8, 13 | 50 |
| Fogel et al. (1984) (114) | | Bupropion | 1, 3, 5, 7, 8, 10, 11, 12, 13 | 69.2 |
| Gwirtsman et al. (1983) (65) | | Doxepin | 1, 3, 4, 5, 6, 7, 8, 13 | 61.5 |
|  |  | Maprotiline |  |  |
| Hebenstreit et al. (1989) (99) | | Paroxetine | 1, 2, 3, 4, 5, 8, 9, 12, 13, 14 | 71.4 |
| Hrdina and Lapierre (1986) (109) | | Maprotiline | 1, 3, 4, 5, 7, 8, 9, 10 | 61.5 |
| Hrdina et al. (1988) (67) | | Doxepin (+ DDOX) | 1, 2, 3, 4, 5, 7, 8, 9, 10, 12 | 71.4 |
| Kelly et al. (1989) (101) | | Fluoxetine (+ DFX) | 1, 3, 4, 5, 6, 7, 8, 9, 10, 11 | 76.9  *continued* |
| Klok et al. (1981) (68) | | Clomipramine (+ DCL) | 1, 3, 5, 6, 7, 8, 9, 12, 13, 14 | 71.4 |
|  |  | Fluvoxamine |  |  |
| Lehmann et al. (1982) (71) | | Amitriptyline (+ NT) | 1, 2, 3, 4, 5, 6, 7, 9, 10, 11, 12, 13 | 92.3 |
|  |  | Nortriptyline |  |  |
| Linnoila et al. (1980) (72) | | Clomipramine  (+ DCL) | 1, 3, 5, 7, 8, 9, 12, 13 | 61.5 |
|  |  | Doxepin (+ DDOX) |  |  |
| Matuzas et al. (1982) (73) | | Imipramine (+ DMI) | 1, 3, 4, 5, 8, 12 | 46.2 |
| McCue et al. (1989) (74) | | Nortriptyline | 1, 2, 3, 4, 5, 7, 8, 13, 14 | 64.3 |
| Mendlewicz et al. (1980) (75) | | Amitriptyline (+ NT) | 1, 3, 5, 6, 7, 8, 13, 14 | 57.1 |
| Montgomery et al. (1983) (77) | | Imipramine (+ DMI) | 1, 2, 3, 4, 5, 6, 8, 10, 11, 12, 13 | 84.6 |
|  |  | Mianserin |  |  |
| Montgomery et al. (1980) (78) | | Amitriptyline (NT) | 1, 2, 3, 4, 5, 6, 7, 8, 10, 11, 12, 13, 14 | 92.9 |
|  |  | Maprotiline | 1, 2, 3, 4, 5, 6, 7, 8, 10, 11, 12, 14 | 85.7 |
| Moyes et al. (1980) (79) | | Amitriptyline (+ NT) | 1, 2, 3, 5, 7, 8, 9, 10, 12 | 69.2 |
|  |  | Clomipramine (+ DCL) | 1, 2, 3, 5, 7, 8, 9, 10, 12, 14 | 71.4 |
| Müller-Oerlinghausen and Fähndrich (1985) (82) | | Clomipramine | 3, 5, 7, 8, 12, 13 | 46.2 |
|  |  | Maprotiline | 1, 3, 5, 7, 8, 12, 13 | 53.8 |
| Robinson et al. (1985) (85) | | Amitriptyline (+ NT) | 1, 2, 4, 5, 6, 8, 12, 13 | 61.5 |
| Simpson et al. (1982) (86) | | Imipramine (+ DMI) | 1, 3, 4, 8, 12 | 38.5 |
| Thomson et al. (1982) (89) | | Amitriptyline (+ NT) | 1, 3, 4, 5, 6, 7, 8, 9, 10, 12, 13 | 84.6 |
| Vandel et al. (1982) (90) | | Clomipramine (+ DCL) | 1, 2, 3, 5, 8, 9, 13 | 53.8 |
| Veith et al. (1983) (91) | | Desipramine | 1, 3, 4, 5, 6, 7, 8, 9, 13 | 69.2 |
|  |  | Amitriptyline (+ NT) |  |  |
| Veith et al. (1980) (92) | | Desipramine | 1, 3, 4, 8, 9, 12, 13, 14 | 57.1 |
| Mean 1980s: 67.0 | | | | |
| 1990s | | | | |
| Birmaher et al. (1998) (45) | | Amitriptyline | 1, 4, 5, 6, 7, 8, 9, 14 | 57.1 |
| DUAG (1990) (58) | | Clomipramine (+ DCL) | 1, 2, 3, 4, 5, 7, 8, 9, 10, 11, 12, 13, 14 | 92.9 |
|  |  | Paroxetine | 1, 2, 3, 4, 5, 7, 8, 9, 10, 12, 13, 14 | 85.7 |
| DUAG (1993) (60) | | Clomipramine (+ DCL) | 1, 2, 3, 4, 5, 6, 7, 8, 9, 10, 11, 12, 13 | 100 |
|  |  | Moclobemide |  |  |
| DUAG (1999) (61) | | Clomipramine (+ DCL) | 1, 2, 3, 4, 5, 6, 7, 8, 9, 10, 12, 13, 14 | 92.9 |
| Geller et al. (1990) (63) | | Nortriptyline | 1, 2, 3, 4, 5, 6, 7, 8, 9, 10, 11, 12, 13 | 100 |
| Geller et al. (1992) (64) | | Nortripytline | 1, 2, 3, 4, 5, 6, 7, 8, 9, 10, 11, 12, 13 | 100 |
| Ghose (1997) (97) | | Paroxetine | 1, 3, 5, 6, 8, 9, 10, 11, 12, 14 | 71.4 |
| Kasper et al. (1993) (100) | | Fluvoxamine | 1, 3, 4, 5, 6, 7, 8, 9, 10, 11, 13, 14 | 85.7 |
|  |  | Maprotiline |  |  |
| Kutcher et al. (1994) (70) | | Desipramine | 3, 4, 5, 6, 7, 8, 9, 10, 11, 12, 13 | 84.6  *continued* |
| Monteleone and Fabrazzo (1994) (76) | | Amitriptyline (+ NT) | 1, 2, 3, 4, 5, 6, 7, 8, 9, 10, 11, 12, 13, 14 | 100 |
|  |  | Mianserin |  |  |
| Nathan et al. (1990) (83) | | Desipramine | 1, 4, 5, 6, 7, 8, 10, 12, 13, 14 | 71.4 |
|  |  | Fluvoxamine | 1, 4, 5, 6, 7, 8, 10, 12, 13 | 69.2 |
| Pollock et al. (1993) (84) | | Clomipramine (+ DCL) | 1, 3, 4, 5, 6, 8, 10, 11, 13, 14 | 71.4 |
| Preskorn et al. (1991) (104) | | Fluoxetine (+ DFX) | 1, 4, 5, 6, 8, 9, 10, 11, 13 | 69.2 |
| Simeon et al. (1990) (108) | | Fluoxetine (+ DFX) | 4, 5, 8, 10, 11 | 38.5 |
| Mean 1990s: 82.9 | | | | |
| 2000s | | | | |
| Allard et al. (2004) (94) | | Citalopram | 4, 5, 6, 7, 8, 9, 12, 13 | 61.5 |
|  |  | Venlafaxine (+ ODV) |  |  |
| Leuchter et al. (2009) (102) | | Escitalopram | 1, 4, 5, 6, 7, 8, 9, 12 | 61.5 |
| Müller et al. (2003) (81) | | Amitriptyline (+ NT) | 1, 2, 3, 4, 5, 6, 8, 13, 14 | 64.3 |
|  |  | Doxepin (+ DDOX) |  |  |
| Perez et al. (2001) (103) | | Fluoxetine (+ DFX) | 1, 2, 3, 4, 5, 6, 7, 8, 10, 11, 12, 13 | 92.3 |
| Reis et al. (2004) (105) | | Sertraline (+ DSERT) | 1, 3, 4, 5, 6, 7, 8, 9, 12, 13, 14 | 78.6 |
|  |  | Paroxetine | 1, 2, 3, 4, 5, 6, 7, 8, 9, 12, 13, 14 | 85.7 |
| Streim et al. (2000) (88) | | Nortriptyline | 1, 2, 3, 4, 5, 6, 7, 8, 9, 10, 11, 13 | 92.3 |
| Mean 2000s: 73.6 | | | | |
| 2010s | | | | |
| Berm et al. (2016) (44) | | Nortriptyline | 4, 5, 6, 7, 8, 9, 12, 13 | 61.5 |
|  |  | Venlafaxine |  |  |
| Breitenstein et al. (2016) (46) | | Amitriptyline | 1, 4, 5, 7, 8, 9, 12, 14 | 57.1 |
|  |  | Amitriptylineoxide |  |  |
|  |  | Nortriptyline |  |  |
|  |  | Trimipramine |  |  |
|  |  | Citalopram |  |  |
|  |  | Escitalopram |  |  |
|  |  | Paroxetine |  |  |
|  |  | Sertraline |  |  |
|  |  | Venlafaxine |  |  |
| Hodgson et al. (2014) (66) | | Nortriptyline | 1, 2, 3, 4, 5, 6, 8, 9, 13 | 69.2 |
|  |  | Escitalopram |  |  |
| Lobello et al. (2010) (112) | | Venlafaxine (+ ODV) | 3, 4, 5, 6, 7, 8, 9, 10, 11 | 69.2 |
| Martiny et al. (2012) (113) | | Venlafaxine (+ ODV) | 1, 3, 4, 5, 6, 7, 8, 12, 13 | 69.2 |
| Sakolsky et al. (2011) (106) | | Citalopram | 1, 3, 4, 5, 7, 8, 9, 13, 14 | 64.3 |
|  |  | Fluoxetine (+ DFX) |  |  |
|  |  | Paroxetine |  |  |
|  |  | Venlafaxine (+ ODV) |  |  |
| Mean 2010s: 61.6 | | | | |

*Note.* DUAG Danish University Antidepressant group, Abbreviations substances: Abbreviations substances: NT Nortriptyline, DDOX N-desmethyldoxepin, DCL N-desmethyl-clomipramine, DMI Desipramine, DFX N-desmethyl-fluoxetine, ODV O-desmethyl-venlafaxine

**Table S8. Additional subgroup analyses**

| Subgroup analyses (N treatment arms for each subgroup) | Chi^2^ | df | p | I^2^ |
| --- | --- | --- | --- | --- |
| Age [Adolescents ≤ 20 years(5) vs. Adults (21)] | 0,01 | 1 | 0,91 | 0% |
| Substance classes [TCA (16) vs. SSRI + SSNRI (9)] | 0,00 | 1 | 0,99 | 0% |
| Quality Assessment (QA) total rating: median split  [$\boldsymbol{\leq}$ Median (17) vs. > Median (10)] | 0,20 | 1 | 0,65 | 0% |
| Publication date [Before 1990 (18) vs. After 1990 (9)] | 0,21 | 1 | 0,65 | 0% |

*Note.* The sum of treatment arms included in the first subgroup analysis does not add up to 27 (N total treatment arms in meta-analysis) since subgroup of older adults did not fulfill the minimum number of three treatment arms

**Figures**

**Identification of studies via other methods**

**Identification of studies via databases and registers**

Records identified from:

Citation searching (n = 3065)

Records removed *before screening*:

Duplicate records removed (n = 603)

Records identified from Pubmed & Web of Science

Databases (n = 1869)

**Identification**

Records excluded**

(n = 3001)

Records excluded**

(n = 1160)

Records screened

(n = 1266)

Reports not retrieved

(n = 0)

Reports not retrieved

(n = 0)

Reports sought for retrieval

(n = 64)

Reports sought for retrieval

(n = 106)

**Screening**

Reports excluded (n = 77)

Study design (n = 24)

Sample characteristics (n = 9)

Medication (n = 3)

Concentration-effect relationship

not reported (n = 40)

Duplicates (n = 1)

Reports excluded: 28

Study design (n = 10)

Sample characteristics (n = 8)

Concentration-effect relationship

not reported (n = 10)

Reports assessed for eligibility

(n = 64)

Reports assessed for eligibility

(n = 106)

Studies included in systematic review

(n = 65)

Studies included in meta-analysis

(n = 19)

**Included**

*Consider, if feasible to do so, reporting the number of records identified from each database or register searched (rather than the total number across all databases/registers).

**If automation tools were used, indicate how many records were excluded by a human and how many were excluded by automation tools.

*From:*  Page MJ, McKenzie JE, Bossuyt PM, Boutron I, Hoffmann TC, Mulrow CD, et al. The PRISMA 2020 statement: an updated guideline for reporting systematic reviews. BMJ 2021;372:n71. doi: 10.1136/bmj.n71. For more information, visit: <http://www.prisma-statement.org/>

**Figure S2. Summarized results from Risk of Bias rating**


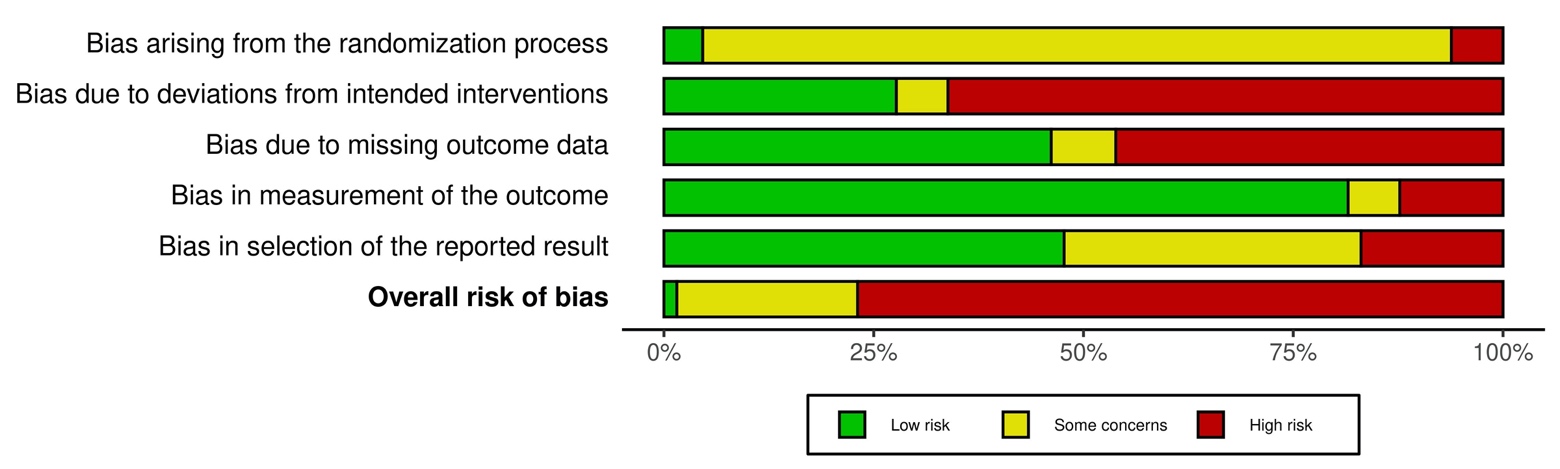


**Figure S3. Funnel plot of 27 treatment arms included in meta-analysis**


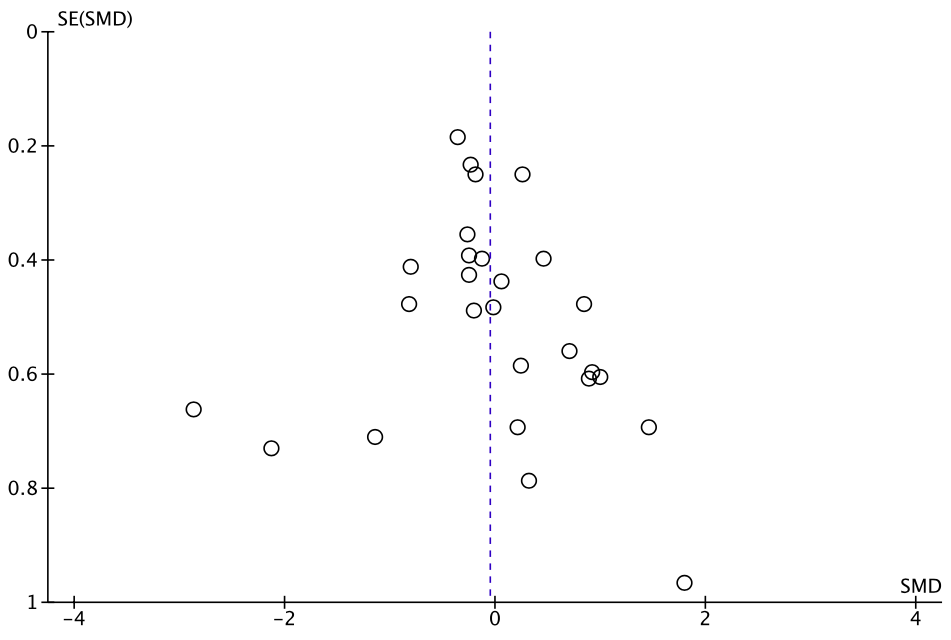


**Figure S4. Results from forced-entry meta-regression including twelve predictors**


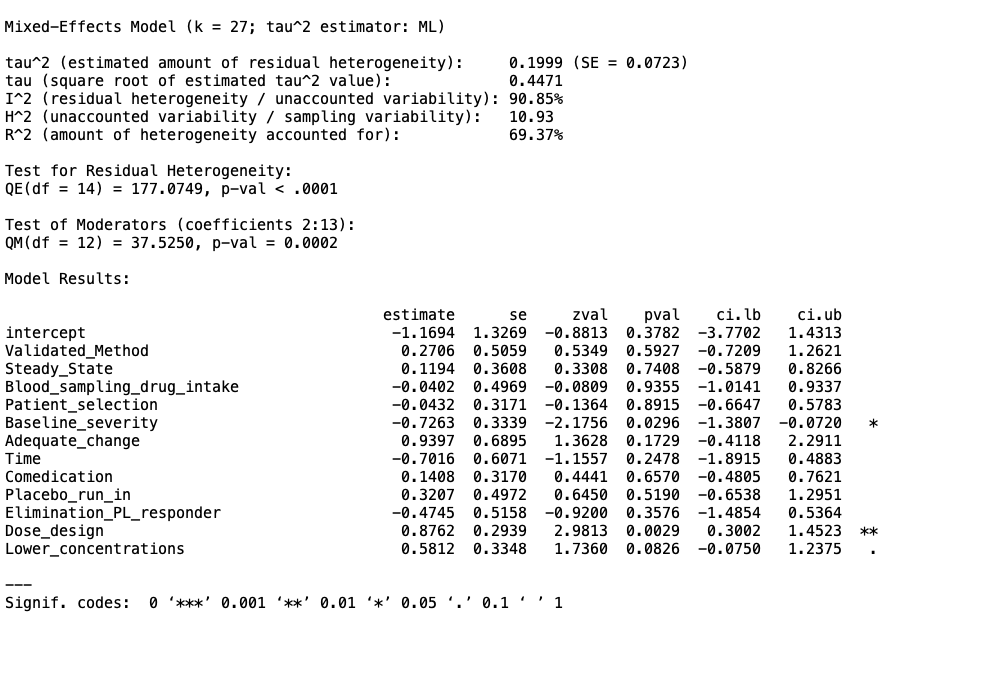


**Figure S5. Results from iterative ANOVAs including quality assessment criteria**


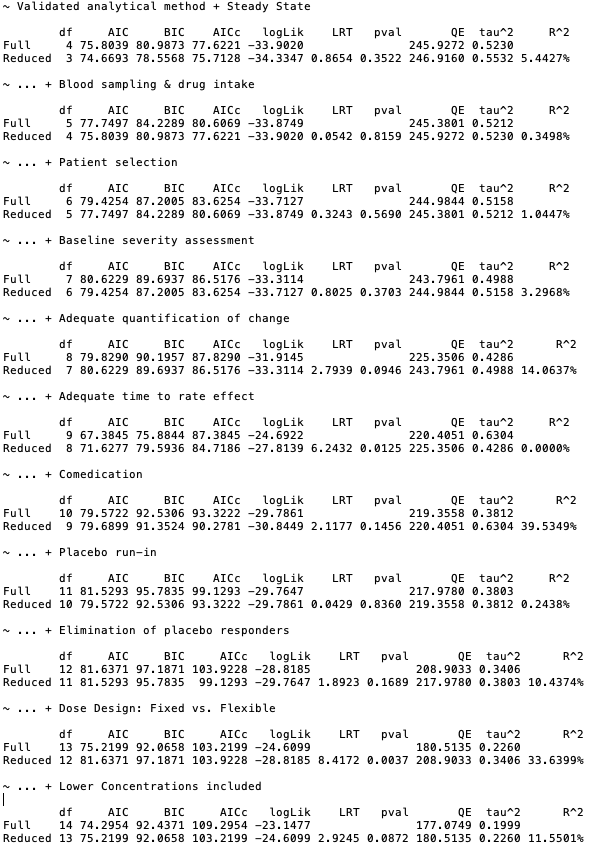


*Note.* “Validated analytical method” was used as baseline predictor.

1. Zernig G, Hiemke C. 'Pharmacokinetic and Pharmacodynamic Principles.' In: Riederer P, Laux G, Nagatsu T, Le W, Riederer C, editors. NeuroPsychopharmacotherapy. Cham: Springer International Publishing (2020). p. 1-19. doi: 10.1007/978-3-319-56015-1_1-1

2. Kloosterboer SM, Vierhout D, Stojanova J, Egberts KM, Gerlach M, Dieleman GC, et al. Psychotropic drug concentrations and clinical outcomes in children and adolescents: a systematic review. Expert Opin Drug Saf (2020) 19(7):873-90. doi: 10.1080/14740338.2020.1770224

3. Protti M, Mandrioli R, Marasca C, Cavalli A, Serretti A, Mercolini L. New-generation, non-SSRI antidepressants: Drug-drug interactions and therapeutic drug monitoring. Part 2: NaSSAs, NRIs, SNDRIs, MASSAs, NDRIs, and others. Med Res Rev. (2020) 40(5):1794-832. doi: 10.1002/med.21671

4. Hiemke C. Concentration-Effect Relationships of Psychoactive Drugs and the Problem to Calculate Therapeutic Reference Ranges. Ther Drug Monit (2019) 41(2):174-9. doi: 10.1097/FTD.000000000000058242

5. Hiemke C, Bergemann N, Clement HW, Conca A, Deckert J, Domschke K, et al. Consensus Guidelines for Therapeutic Drug Monitoring in Neuropsychopharmacology: Update 2017. Pharmacopsychiatry (2018) 51(1-02):e1. doi: 10.1055/s-0037-1600991

6. Mandrioli R, Protti M, Mercolini L. New-Generation, Non-SSRI Antidepressants: Therapeutic Drug Monitoring and Pharmacological Interactions. Part 1: SNRIs, SMSs, SARIs. Curr Med Chem. (2018) 25(7):772-92. doi: 10.2174/0929867324666170712165042

7. Grundmann M, Kacirova I, Urinovska R. Therapeutic monitoring of psychoactive drugs - antidepressants: a review. Biomed Pap Med Fac Univ Palacky Olomouc Czech Repub (2015) 159(1):35-43. doi: 10.5507/bp.2013.020

8. Lloret-Linares C, Bellivier F, Haffen E, Aubry JM, Daali Y, Heron K, et al. Markers of Individual Drug Metabolism: Towards the Development of a Personalized Antidepressant Prescription. Curr Drug Metab (2015) 16(1):17-45. doi: 10.2174/138920021601150702160728

9. Hefner G, Laib AK, Sigurdsson H, Hohner M, Hiemke C. The value of drug and metabolite concentration in blood as a biomarker of psychopharmacological therapy. Int Rev Psychiatry. (2013) 25(5):494-508. doi: 10.3109/09540261.2013.836475

10. Mandrioli R, Mercolini L, Saracino MA, Raggi MA. Selective serotonin reuptake inhibitors (SSRIs): therapeutic drug monitoring and pharmacological interactions. Curr Med Chem. (2012) 19(12):1846-63. doi: 10.2174/092986712800099749

11. Grunder G, Hiemke C, Paulzen M, Veselinovic T, Vernaleken I. Therapeutic plasma concentrations of antidepressants and antipsychotics: lessons from PET imaging. Pharmacopsychiatry. (2011) 44(6):236-48. doi: 10.1055/s-0031-1286282

12. Pichini S, Papaseit E, Joya X, Vall O, Farre M, Garcia-Algar O, et al. Pharmacokinetics and therapeutic drug monitoring of psychotropic drugs in pediatrics. Ther Drug Monit. (2009) 31(3):283-318. doi: 10.1097/FTD.0b013e31819f3328

13. Hiemke C. Therapeutic drug monitoring in neuropsychopharmacology: does it hold its promises? Eur Arch Psychiatry Clin Neurosci. (2008) 258 Suppl 1:21-7. 10.1007/s00406-007-1005-y

14. Wille SM, Cooreman SG, Neels HM, Lambert WE. Relevant issues in the monitoring and the toxicology of antidepressants. Crit Rev Clin Lab Sci. (2008) 45(1):25-89. doi: 10.1080/10408360701713112

15. Hiemke C. Clinical utility of drug measurement and pharmacokinetics: therapeutic drug monitoring in psychiatry. Eur J Clin Pharmacol (2008) 64(2):159-66. doi: 10.1007/s00228-007-0430-1

16. Laux G, Baumann P, Hiemke C. 'Therapeutic drug monitoring of antidepressants — clinical aspects' In: Gerlach, M, Deckert, J, Double, K, Koutsilieri, E, editors. Neuropsychiatric Disorders - An Integrative Approach. Vienna: Springer Vienna (2007). p. 261-286 https://link.springer.com/book/10.1007/978-3-211-73574-9?page=2#toc

17. DeVane CL, Stowe ZN, Donovan JL, Newport DJ, Pennell PB, Ritchie JC, et al. Therapeutic drug monitoring of psychoactive drugs during pregnancy in the genomic era: challenges and opportunities. J Psychopharmacol. (2006) 20(4 Suppl):54-9. doi: 10.1177/1359786806066054

18. Hendset M, Haslemo T, Rudberg I, Refsum H, Molden E. The complexity of active metabolites in therapeutic drug monitoring of psychotropic drugs. Pharmacopsychiatry. (2006) 39(4):121-7. doi: 10.1055/s-2006-946701

19. Eap CB, Jaquenoud Sirot E, Baumann P. Therapeutic monitoring of antidepressants in the era of pharmacogenetics studies. Ther Drug Monit. (2004) 26(2):152-5. doi: 10.1097/00007691-200404000-00011

20. Bengtsson F. Therapeutic drug monitoring of psychotropic drugs. TDM "nouveau". Ther Drug Monit. (2004) 26(2):145-51. doi: 10.1097/00007691-200404000-00010

21. Mitchell PB. Therapeutic drug monitoring of non-tricyclic antidepressant drugs. Clin Chem Lab Med (2004) 42(11):1212-8. doi: 10.1515/CCLM.2004.243

22. Baumann P, Hiemke C, Ulrich S, Eckermann G, Gaertner I, Gerlach M, et al. The AGNP-TDM expert group consensus guidelines: therapeutic drug monitoring in psychiatry. Pharmacopsychiatry. (2004) 37(6):243-65. doi: 10.1055/s-2004-832687

23. Ulrich S, Läuter J. Comprehensive survey of the relationship between serum concentration and therapeutic effect of amitriptyline in depression. Clin Pharmacokinet (2002) 41(11):853-76. doi: 10.2165/00003088-200241110-00004.

24. Mitchell PB. Therapeutic drug monitoring of psychotropic medications. Br J Clin Pharmacol. (2001) 52 Suppl 1:45S-54S. doi: 10.1046/j.1365-2125.2001.0520s1045.x

25. Rasmussen BB, Brosen K. Is therapeutic drug monitoring a case for optimizing clinical outcome and avoiding interactions of the selective serotonin reuptake inhibitors? Ther Drug Monit. (2000) 22(2):143-54. doi: 10.1097/00007691-200004000-00001

26. Burke MJ, Preskorn SH. Therapeutic drug monitoring of antidepressants: cost implications and relevance to clinical practice. Clin Pharmacokinet. (1999) 37(2):147-65. doi: 10.2165/00003088-199937020-00004

27. Balant-Gorgia E, Balant L. Therapeutic Drug Monitoring: Relevance During the DRug Treatment of Psychiatric Disorders. Drug Ther CNS Drug. (1995) (4):423-355. doi: 10.2165/00023210-199504060-00006

28. Preskorn SH, Burke MJ, Fast GA. Therapeutic drug monitoring. Principles and practice. Psychiatr Clin North Am. (1993) 16(3):611-45. doi: <https://doi.org/10.1016/S0193-953X(18)30167-9>

29. Perry PJ, Pfohl BM, Holstad SG. The relationship between antidepressant response and tricyclic antidepressant plasma concentrations. A retrospective analysis of the literature using logistic regression analysis. Clin Pharmacokinet. (1987) 13(6):381-92. doi: 10.2165/00003088-198713060-00003

30. DeVane CL. Monitoring cyclic antidepressants. Clin Lab Med. (1987) 7(3):551-66.

31. Tricyclic antidepressants--blood level measurements and clinical outcome: an APA Task Force report. Task Force on the Use of Laboratory Tests in Psychiatry. Am J Psychiatry. (1985) 142(2):155-62. doi: [10.1176/ajp.142.2.155](https://doi.org/10.1176/ajp.142.2.155)

32. Gualtieri CT, Golden R, Evans RW, Hicks RE. Blood level measurement of psychoactive drugs in pediatric psychiatry. Ther Drug Monit. (1984) 6(2):127-41. doi: 10.1097/00007691-198406000-00001

33. Van Brunt N. The clinical utility of tricyclic antidepressant blood levels: a review of the literature. Ther Drug Monit. (1983) 5(1):1-10. doi: 10.1097/00007691-198303000-00001

34. Norman TR, Maguire KP, Scoggins BA, Burrows GD. Monitoring and interpretation of antidepressant plasma concentrations. Aust N Z J Psychiatry. (1982) 16(1):74-8. doi: 10.3109/00048678209159473

35. Risch SC, Kalin NH, Janowsky DS, Huey LY. Indications and guidelines for plasma tricyclic antidepressant concentration monitoring. J Clin Psychopharmacol. (1981) 1(2):59-63.

36. Levine RR. The role of plasma concentrations in the use of tricyclic antidepressant drugs. Prog Neuropsychopharmacol. (1979) 3(1-3):211-22. doi: 10.1016/0364-7722(79)90085-7

37. Sjoqvist F. Monitoring of antidepressant drug plasma levels: the next ten years. Prog Neuropsychopharmacol. (1979) 3(1-3):201-10. doi: 10.1016/0364-7722(79)90084-5

38. Orsulak PJ, Schildkraut JJ. Guidelines for therapeutic monitoring of tricyclic antidepressant plasma levels. Ther Drug Monit. (1979) 1(2):199-208. doi: 10.1097/00007691-197901020-00002

39. Kragh-Sørensen P, Hansen CE, Baastrup E. Relationship between antidepressant effect and plasma level of nortriptyline. Clinical studies. Tricyclic Antidepressants. (1978):77. doi: 10.1055/s-0028-1094474

40. Gram LF. Plasma level monitoring of tricyclic antidepressant therapy. Clin Pharmacokinet. (1977) 2(4):237-51. doi: 10.2165/00003088-197702040-00001

41. Gelenberg AJ, Freeman MP, Markowitz JC, Rosenbaum JF, Thase ME, Trivedi MH, et al. Practice guideline for the treatment of patients with major depressive disorder third edition. The American journal of psychiatry. (2010);167(10):1. http://www.psychiatryonline.com/pracGuide/pracGuideTopic_7.aspx.

42. DGPPN, BÄK, KBV, AWMF (Hrsg.) für die Leitliniengruppe Unipolare Depression*. S3-Leitlinie/Nationale Versor- gungsLeitlinie Unipolare Depression – Langfassung, 2. Auflage. Version 5. 2015 [cited: 2022-10-01]. DOI: 10.6101/AZQ/000364. www.depression.versorgungsleitlinien.de.

43. Amin MM, Cooper R, Khalid R, Lehmann HE. A comparison of desipramine and amitriptyline plasma levels and therapeutic response. Psychopharmacol Bull. (1978) 14(1):45-6.

44. Berm E, Kok R, Hak E, Wilffert B. Relation between CYP2D6 Genotype, Phenotype and Therapeutic Drug Concentrations among Nortriptyline and Venlafaxine Users in Old Age Psychiatry. Pharmacopsychiatry. (2016) 49(5):186-90. doi: 10.1055/s-0042-105443

45. Birmaher B, Waterman GS, Ryan ND, Perel J, McNabb J, Balach L, et al. Randomized, controlled trial of amitriptyline versus placebo for adolescents with "treatment-resistant" major depression. J Am Acad Child Adolesc Psychiatry. (1998) 37(5):527-35. doi: 10.1097/00004583-199805000-00015

46. Breitenstein B, Scheuer S, Bruckl TM, Meyer J, Ising M, Uhr M, et al. Association of ABCB1 gene variants, plasma antidepressant concentration, and treatment response: Results from a randomized clinical study. J Psychiatr Res. (2016) 73:86-95. doi: 10.1016/j.jpsychires.2015.11.010

47. Fähndrich E. Das AMDP-System: Manual zur Dokumentation Psychiatrischer Befunde, third ed. Berlin: Springer (1979).

48. Breyer-Pfaff U, Giedke H, Gaertner HJ, Nill K. Validation of a therapeutic plasma level range in amitriptyline treatment of depression. J Clin Psychopharmacol. (1989) 9(2):116-21. doi: 10.1097/00004714-198904000-00008

49. Brunswick DJ, Amsterdam JD, Potter L, Caroff S, Rickels K. Relationship between tricyclic antidepressant plasma levels and clinical response in patients treated with desipramine or doxepin. Acta Psychiatr Scand. (1983) 67(6):371-7. doi: 10.1111/j.1600-0447.1983.tb09717.x

50. Burch JE, Ahmed O, Hullin RP, Mindham RH. Antidepressive effect of amitriptyline treatment with plasma drug levels controlled within three different ranges. Psychopharmacology (Berl). (1988) 94(2):197-205. doi: 10.1007/BF00176845

51. Burrows GD, Maguire KP, Scoggins BA, Stevenson J, Davies B. Plasma nortriptyline and clinical response--a study using changing plasma levels. Psychol Med. (1977) 7(1):87-91. doi: 10.1017/s0033291700023163

52. Dahl LE, Lundin L, le Fevre Honore P, Dencker SJ. Antidepressant effect of femoxetine and desipramine and relationship to the concentration of amine metabolites in cerebrospinal fluid. A double-blind evaluation. Acta Psychiatr Scand. (1982) 66(1):9-17. doi: 10.1111/j.1600-0447.1982.tb00909.x

53. Gurney C, Roth M, Garside RF, Kerr TA, Schapira K. Studies in the classification of affective disorders. The relationship between anxiety states and depressive illnesses. II. Br J Psychiatry. (1972) 121(561):162-6. doi: 10.1192/bjp.121.2.162

54. De Wilde JE, Doogan DP. Fluvoxamine and chlorimipramine in endogenous depression. J Affect Disord. (1982) 4(3):249-59. doi: 10.1016/0165-0327(82)90009-x

55. De Wilde JE, Mertens C, Wakelin JS. Clinical trials of fluvoxamine vs chlorimipramine with single and three times daily dosing. Br J Clin Pharmacol. (1983) 15 Suppl 3:427S-31S. doi: 10.1111/j.1365-2125.1983.tb02133.x

56. DUAG. Citalopram: clinical effect profile in comparison with clomipramine. A controlled multicenter study. Danish University Antidepressant Group. Psychopharmacology (Berl). (1986) 90(1):131-8. doi: 10.1007/BF00172884

57. Gurney C. Diagnostic scales for affective disorders. [Conference presentation] Proceedings of the Fifth World Conference of Psychiatry, Mexico City. (1971).

58. DUAG. Paroxetine: a selective serotonin reuptake inhibitor showing better tolerance, but weaker antidepressant effect than clomipramine in a controlled multicenter study. Danish University Antidepressant Group. J Affect Disord. (1990) 18(4):289-99. doi: 10.1016/0165-0327(90)90081-i

59. Lingjaerde O, Ahlfors U, Bech P, Dencker S, Elgen K. The UKU side effect rating scale: a new comprehensive rating scale for psychotropic drugs and a cross-sectional study of side effects in neuroleptic-treated patients. Acta Psychiatrica Scandinavica. (1987). doi: [10.1111/j.1600-0447.1987.tb10566.x](https://psycnet.apa.org/doi/10.1111/j.1600-0447.1987.tb10566.x)

60. DUAG. Moclobemide: a reversible MAO-A-inhibitor showing weaker antidepressant effect than clomipramine in a controlled multicenter study. Danish University Antidepressant Group. J Affect Disord. (1993) 28(2):105-16. doi: 10.1016/0165-0327(93)90039-m

61. DUAG. Clomipramine dose-effect study in patients with depression: clinical end points and pharmacokinetics. Danish University Antidepressant Group (DUAG). Clin Pharmacol Ther. (1999) 66(2):152-65. doi: 10.1016/S0009-9236(99)90053-X

62. Feet PO, Larsen S, Lillevold PE, Liden A, Holm V, Robak OH. Comparison of the serum levels in primary non-agitated depressed out-patients treated with imipramine in combination with placebo, diazepam or dixyrazine. Acta Psychiatr Scand. (1987) 75(4):435-40. doi: 10.1111/j.1600-0447.1987.tb02812.x

63. Geller B, Cooper TB, Graham DL, Marsteller FA, Bryant DM. Double-blind placebo-controlled study of nortriptyline in depressed adolescents using a "fixed plasma level" design. Psychopharmacol Bull. (1990) 26(1):85-90.

64. Geller B, Cooper TB, Graham DL, Fetner HH, Marsteller FA, Wells JM. Pharmacokinetically designed double-blind placebo-controlled study of nortriptyline in 6- to 12-year-olds with major depressive disorder. J Am Acad Child Adolesc Psychiatry. (1992) 31(1):34-44. doi: 10.1097/00004583-199201000-00007

65. Gwirtsman HE, Ahles S, Halaris A, DeMet E, Hill MA. Therapeutic superiority of maprotiline versus doxepin in geriatric depression. J Clin Psychiatry. (1983) 44(12):449-53.

66. Hodgson K, Tansey K, Dernovsek MZ, Hauser J, Henigsberg N, Maier W, et al. Genetic differences in cytochrome P450 enzymes and antidepressant treatment response. J Psychopharmacol. (2014) 28(2):133-41. doi: 10.1177/0269881113512041

67. Hrdina PD, Lapierre YD, Horn E, Bakish D, Browne M. Antidepressant plasma levels and clinical response in depressed patients treated with oxaprotiline and doxepin. Int Clin Psychopharmacol. (1988) 3(3):205-14. doi: 10.1097/00004850-198807000-00002

68. Klok CJ, Brouwer GJ, van Praag HM, Doogan D. Fluvoxamine and clomipramine in depressed patients. A double-blind clinical study. Acta Psychiatr Scand. (1981) 64(1):1-11. doi: 10.1111/j.1600-0447.1981.tb00756.x

69. Kragh-Sorensen P, Hansen CE, Baastrup PC, Hvidberg EF. Self-inhibiting action of nortriptylin's antidepressive effect at high plasma levels: a randomized double-blind study controlled by plasma concentrations in patients with endogenous depression. Psychopharmacologia. (1976) 45(3):305-12. doi: 10.1007/BF00421145

70. Kutcher S, Boulos C, Ward B, Marton P, Simeon J, Ferguson HB, et al. Response to desipramine treatment in adolescent depression: a fixed-dose, placebo-controlled trial. J Am Acad Child Adolesc Psychiatry. (1994) 33(5):686-94. doi: 10.1097/00004583-199406000-00010

71. Lehmann LS, Bowden CL, Redmond FC, Stanton BC. Amitriptyline and nortriptyline response profiles in unipolar depressed patients. Psychopharmacology (Berl). (1982) 77(2):193-7. doi: 10.1007/BF00431947

72. Linnoila M, Seppala T, Mattila MJ, Vihko R, Pakarinen A, Skinner T, 3rd. Clomipramine and doxepin in depressive neurosis. Plasma levels and therapeutic response. Arch Gen Psychiatry. (1980) 37(11):1295-9. doi: 10.1001/archpsyc.1980.01780240093011

73. Matuzas W, Javaid JI, Glass R, Davis JM, Ross JA, Uhlenhuth EH. Plasma concentrations of imipramine and clinical response among depressed outpatients. J Clin Psychopharmacol. (1982) 2(2):140-2.

74. McCue RE, Georgotas A, Suckow RF, Cooper TB. 10-Hydroxynortriptyline and treatment effects in elderly depressed patients. J Neuropsychiatry Clin Neurosci. (1989) 1(2):176-80. doi: 10.1176/jnp.1.2.176

75. Mendlewicz J, Linkowski P, Rees JA. A double-blind comparison of dothiepin and amitriptyline in patients with primary affective disorder: serum levels and clinical response. Br J Psychiatry. (1980) 136:154-60. doi: 10.1192/bjp.136.2.154

76. Monteleone P, Fabrazzo M. Blood levels of mianserin and amitriptyline and clinical response in aged depressed patients. Pharmacopsychiatry. (1994) 27(6):238-41. doi: 10.1055/s-2007-1014311

77. Montgomery SA, Roy D, Wynne-Willson S, Robinson C, Montgomery DB. Plasma levels and clinical response with imipramine in a study comparing efficacy with mianserin and nomifensine. Br J Clin Pharmacol. (1983) 15 Suppl 2:205S-11S. doi: 10.1111/j.1365-2125.1983.tb05867.x

78. Montgomery SA, McAuley R, Montgomery DB, Dawling S, Braithwaite RA. Pharmacokinetics and efficacy of maprotiline and amitriptyline in endogenous depression: a double-blind controlled trial. Clin Ther. (1980) 3(4):292-310. doi: [10.1111/j.1600-0447.1981.tb00735.x](https://doi.org/10.1111/j.1600-0447.1981.tb00735.x)

79. Moyes IC, Ray RL, Moyes RB. Plasma levels and clinical improvement--a comparative study of clomipramine and amitriptyline in depression. Postgrad Med J. (1980) 56 Suppl 1:127-9.

80. Mulgirigama LD, Pare CM, Turner P, Wadsworth J, Witts DJ. Clinical responses in depressed patients in relation to plasma levels of tricyclic antidepressants and tyramine pressor response. Postgrad Med J. (1977) 53 Suppl 4:155-9.

81. Müller MJ, Dragicevic A, Fric M, Gaertner I, Grasmader K, Hartter S, et al. Therapeutic drug monitoring of tricyclic antidepressants: how does it work under clinical conditions? Pharmacopsychiatry. (2003) 36(3):98-104. doi: 10.1055/s-2003-39983

82. Müller-Oerlinghausen B, Fähndrich E. The relationship between pharmacokinetic data and the clinical response in patients treated with maprotiline or clomipramine by intravenous infusion. Pharmacopsychiatry. (1985) 18(01):100-1. doi: 10.1055/s-2007-1017328

83. Nathan RS, Perel JM, Pollock BG, Kupfer DJ. The role of neuropharmacologic selectivity in antidepressant action: fluvoxamine versus desipramine. J Clin Psychiatry. (1990) 51(9):367-72.

84. Pollock BG, Perel JM, Kupfer DJ, Bowler KA, Miewald JM. Early response patterns associated with successful clomipramine treatment. J Clin Psychopharmacol. (1993) 13(6):442-7. doi: [10.1097/00004714-199312000-00011](https://psycnet.apa.org/doi/10.1097/00004714-199312000-00011)

85. Robinson DS, Cooper TB, Howard D, Corcella J, Albright D. Amitriptyline and hydroxylated metabolite plasma levels in depressed outpatients. J Clin Psychopharmacol. (1985) 5(2):83-8. doi: 10.1097/00004714-198504000-00005

86. Simpson GM, White KL, Boyd JL, Cooper TB, Halaris A, Wilson IC, et al. Relationship between plasma antidepressant levels and clinical outcome for inpatients receiving imipramine. Am J Psychiatry. (1982) 139(3):358-60. doi: 10.1176/ajp.139.3.358

87. Zung WW. A Self-Rating Depression Scale. Arch Gen Psychiatry. (1965) 12:63-70. doi: 10.1001/archpsyc.1965.01720310065008

88. Streim JE, Oslin DW, Katz IR, Smith BD, DiFilippo S, Cooper TB, et al. Drug treatment of depression in frail elderly nursing home residents. Am J Geriatr Psychiatry. (2000) 8(2):150-9. doi: [10.1097/00019442-200005000-00010](https://doi.org/10.1097/00019442-200005000-00010)

89. Thomson J, Rankin H, Ashcroft GW, Yates CM, McQueen JK, Cummings SW. The treatment of depression in general practice: a comparison of L-tryptophan, amitriptyline, and a combination of L-tryptophan and amitriptyline with placebo. Psychol Med. (1982) 12(4):741-51. doi: 10.1017/s0033291700049047

90. Vandel B, Vandel S, Jounet JM, Allers G, Volmat R. Relationship between the plasma concentration of clomipramine and desmethylclomipramine in depressive patients and the clinical response. Eur J Clin Pharmacol. (1982) 22(1):15-20. doi: 10.1007/BF00606419

91. Veith RC, Bielski RJ, Bloom V, Fawcett JA, Narasimhachari N, Friedel RO. Urinary MHPG excretion and treatment with desipramine or amitriptyline: prediction of response, effect of treatment, and methodological hazards. J Clin Psychopharmacol. (1983) 3(1):18-27.

92. Veith RC, Friedel RO, Bloom V, Bielski R. Electrocardiogram changes and plasma desipramine levels during treatment of depression. Clin Pharmacol Ther. (1980) 27(6):796-802. doi: 10.1038/clpt.1980.113

93. Ziegler VE, Clayton PJ, Biggs JT. A comparison study of amitriptyline and nortriptyline with plasma levels. Arch Gen Psychiatry. (1977) 34(5):607-12. doi: 10.1001/archpsyc.1977.01770170117012

94. Allard P, Gram L, Timdahl K, Behnke K, Hanson M, Sogaard J. Efficacy and tolerability of venlafaxine in geriatric outpatients with major depression: a double-blind, randomised 6-month comparative trial with citalopram. Int J Geriatr Psychiatry. (2004) 19(12):1123-30. doi: 10.1002/gps.1190

95. Bjerkenstedt L, Flyckt L, Overo KF, Lingjaerde O. Relationship between clinical effects, serum drug concentration and serotonin uptake inhibition in depressed patients treated with citalopram. A double-blind comparison of three dose levels. Eur J Clin Pharmacol. (1985) 28(5):553-7. doi: 10.1007/BF00544066

96. De Wilde J, Mertens C, Overo KF, Petersen HE. Citalopram versus mianserin. A controlled, double-blind trial in depressed patients. Acta Psychiatr Scand. (1985) 72(1):89-96. doi: 10.1111/j.1600-0447.1985.tb02576.x

97. Ghose K. Tolerance and side-effects of paroxetine in elderly depressed patients. Arch Gerontol Geriatr. (1997) 24(1):35-45. doi: 10.1016/s0167-4943(96)00725-x

98. Ghose K. Lithium salts: therapeutic and unwanted effects. Br J Hosp Med. (1977) 18(6):578-83.

99. Hebenstreit GF, Fellerer K, Zochling R, Zentz A, Dunbar GC. A pharmacokinetic dose titration study in adult and elderly depressed patients. Acta Psychiatr Scand Suppl. (1989) 350:81-4. doi: 10.1111/j.1600-0447.1989.tb07178.x

100. Kasper S, Dotsch M, Kick H, Vieira A, Moller HJ. Plasma concentrations of fluvoxamine and maprotiline in major depression: implications on therapeutic efficacy and side effects. Eur Neuropsychopharmacol. (1993) 3(1):13-21. doi: 10.1016/0924-977x(93)90290-3

101. Kelly MW, Perry PJ, Holstad SG, Garvey MJ. Serum fluoxetine and norfluoxetine concentrations and antidepressant response. Ther Drug Monit. (1989) 11(2):165-70. doi: 10.1097/00007691-198903000-00008

102. Leuchter AF, Cook IA, Marangell LB, Gilmer WS, Burgoyne KS, Howland RH, et al. Comparative effectiveness of biomarkers and clinical indicators for predicting outcomes of SSRI treatment in Major Depressive Disorder: results of the BRITE-MD study. Psychiatry Res. (2009) 169(2):124-31. doi: 10.1016/j.psychres.2009.06.004

103. Perez V, Puiigdemont D, Gilaberte I, Alvarez E, Artigas F, Grup de Recerca en Trastorns A. Augmentation of fluoxetine's antidepressant action by pindolol: analysis of clinical, pharmacokinetic, and methodologic factors. J Clin Psychopharmacol. (2001) 21(1):36-45. doi: 10.1097/00004714-200102000-00008

104. Preskorn SH, Silkey B, Beber J, Dorey C. Antidepressant response and plasma concentrations of fluoxetine. Annals of clinical psychiatry. (1991) 3(2):147-51. doi: [10.3109/10401239109147984](https://doi.org/10.3109/10401239109147984)

105. Reis M, Aberg-Wistedt A, Agren H, Hoglund P, Akerblad AC, Bengtsson F. Serum disposition of sertraline, N-desmethylsertraline and paroxetine: a pharmacokinetic evaluation of repeated drug concentration measurements during 6 months of treatment for major depression. Hum Psychopharmacol. (2004) 19(5):283-91. doi: 10.1002/hup.599

106. Sakolsky DJ, Perel JM, Emslie GJ, Clarke GN, Wagner KD, Vitiello B, et al. Antidepressant exposure as a predictor of clinical outcomes in the Treatment of Resistant Depression in Adolescents (TORDIA) study. J Clin Psychopharmacol. (2011) 31(1):92-7. doi: 10.1097/JCP.0b013e318204b117

107. Klein R, Abikoff H, Barkley R, Campbell M, Leckman J, Ryan N, et al. Clinical trials in children and adolescents. Clinical evaluation of psychotropic drugs: Principles and guidelines. New York: Raven Press (1994). p. 501-46.

108. Simeon JG, Dinicola VF, Ferguson HB, Copping W. Adolescent depression: a placebo-controlled fluoxetine treatment study and follow-up. Prog Neuropsychopharmacol Biol Psychiatry. (1990) 14(5):791-5. doi: 10.1016/0278-5846(90)90050-q

109. Hrdina PD, Lapierre YD. Plasma levels of maprotiline and zimelidine and their relationship to clinical response in depressed patients. Ther Drug Monit. (1986) 8(4):400-6. doi: 10.1097/00007691-198612000-00003

110. Miller PI, Beaumont G, Seldrup J, John V, Luscombe DK, Jones R. Efficacy, side-effects, plasma and blood levels of maprotiline (Ludiomil). J Int Med Res. (1977) 5 Suppl 4:101-11.

111. Montgomery S, McAuley R, Montgomery DB. Relationship between mianserin plasma levels and antidepressant effect in a double-blind trial comparing a single night-time and divided daily dose regimens. Br J Clin Pharmacol. (1978) 5 Suppl 1:71S-6S.

112. Lobello KW, Preskorn SH, Guico-Pabia CJ, Jiang Q, Paul J, Nichols AI, et al. Cytochrome P450 2D6 phenotype predicts antidepressant efficacy of venlafaxine: a secondary analysis of 4 studies in major depressive disorder. J Clin Psychiatry. (2010) 71(11):1482-7. doi: 10.4088/JCP.08m04773blu

113. Martiny K, Lunde M, Bech P, Plenge P. A short-term double-blind randomized controlled pilot trial with active or placebo pindolol in patients treated with venlafaxine for major depression. Nord J Psychiatry. (2012) 66(3):147-54. doi: 10.3109/08039488.2012.674553

114. Fogel P, Mamer OA, Chouinard G, Farrell PG. Determination of plasma bupropion and its relationship to therapeutic effect. Biomed Mass Spectrom. (1984) 11(12):629-32. doi: 10.1002/bms.1200111207

115. Spitzer RL, Endicott J, Williams JB. Research diagnostic criteria. Arch Gen Psychiatry. (1979) 36(12):1381-3. doi: 10.1001/archpsyc.1979.01780120111013

116. Asberg M, Cronholm B, Sjoqvist F, Tuck D. Correlation of subjective side effects with plasma concentrations of nortriptyline. Br Med J. (1970) 4(5726):18-21. doi: 10.1136/bmj.4.5726.18

117. Guy W, Bonato R. Manual for the ECDEU assessment battery, Chevy Chase. MD: National Institute of Mental Health (1970).

118. Hamilton M. A rating scale for depression. J Neurol Neurosurg Psychiatry. (1960) 23:56-62. doi: 10.1136/jnnp.23.1.56

119. Guy W. ECDEU assessment manual for psychopharmacology: US Department of Health, Education, and Welfare, Public Health Service. (1976).

120. Montgomery SA, Asberg M. A new depression scale designed to be sensitive to change. Br J Psychiatry. (1979) 134:382-9. doi: 10.1192/bjp.134.4.382

121. Kellner R. The brief depression rating scale. Assessment of depression: Springer (1986). p. 179-87. doi: 10.1007/978-3-642-70486-4_17

122. Beck AT, Ward CH, Mendelson M, Mock J, Erbaugh J. An inventory for measuring depression. Arch Gen Psychiatry. (1961) 4:561-71. doi: 10.1001/archpsyc.1961.01710120031004

123. Shaffer D, Gould MS, Brasic J, Ambrosini P, Fisher P, Bird H, et al. A children's global assessment scale (CGAS). Arch Gen Psychiatry. (1983) 40(11):1228-31. doi: 10.1001/archpsyc.1983.01790100074010

124. Poznanski EO, Cook SC, Carroll BJ. A depression rating scale for children. Pediatrics. (1979) 64(4):442-50.

125. Derogatis LR, Lipman RS, Rickels K, Uhlenhuth EH, Covi L. The Hopkins Symptom Checklist (HSCL). A measure of primary symptom dimensions. Mod Probl Pharmacopsychiatry. (1974) 7(0):79-110. doi: 10.1159/000395070

126. Cronholm B, Schalling D, Åsberg M. Development of a rating scale for depressive illness. Psychological measurements in psychopharmacology. 7: Karger Publishers (1974). p. 139-50.

127. Sterne JAC, Savovic J, Page MJ, Elbers RG, Blencowe NS, Boutron I, et al. RoB 2: a revised tool for assessing risk of bias in randomised trials. BMJ. (2019) 366:l4898. doi: 10.1136/bmj.l4898
